# Ethno-veterinary uses of Poaceae in Punjab, Pakistan

**DOI:** 10.1371/journal.pone.0241705

**Published:** 2020-11-03

**Authors:** Muhammad Majeed, Khizar Hayat Bhatti, Muhammad Shoaib Amjad, Arshad Mehmood Abbasi, Rainer W. Bussmann, Fahim Nawaz, Audil Rashid, Ansar Mehmood, Majid Mahmood, Wisal Muhammad Khan, Khawaja Shafique Ahmad

**Affiliations:** 1 Department of Botany, University of Gujrat, Hafiz Hayat Campus, Gujrat, Punjab, Pakistan; 2 Department of Botany, Women University of Azad Jammu and Kashmir, Bagh, AJK, Pakistan; 3 Department of Environmental Sciences, COMSATS University Islamabad, Abbottabad Campus, Pakistan; 4 Department of Ethnobotany, Institute of Botany, Ilia State University, La Paz, Bolivia; 5 Department of Agronomy, MNS, University of Agriculture, Multan, Pakistan; 6 Department of Botany, University of Poonch Rawalakot (UPR), Azad Jammu and Kashmir, Pakistan; 7 Department of Zoology, University of Poonch Rawalakot (UPR), Azad Jammu and Kashmir, Pakistan; 8 Department of Botany, Islamia College Peshawar, Peshawar, Pakistan; University of Lincoln, UNITED KINGDOM

## Abstract

Plant species of the Poaceae family are not only used as fodder and forage but also contribute substantially to the treatment of various health disorders, particularly in livestock. Consequently, the present study was aimed to document the therapeutic uses of Poaceae practiced by the inhabitants of the Punjab Province for the treatment of various veterinary health disorders. Semi structured interviews, group discussion and field walks were conducted to collect the data. Quantitative indices including cultural significance index (CSI), relative frequency of citations (RFC), fidelity level (FL), relative popularity level (RPL), and Jaccard Index (JI) were used for the data analysis. Traditional uses of 149 species belonging to 60 genera and 16 tribes of 5 sub families of Poaceae were recorded. Whole plants and leaves were the most consistently used parts with 40.94 and 29.53%. The plants were mainly given orally as fodder (59 reports) without processing followed by decoction (35 reports). Most of the species were employed to treat infectious diseases (25.93%), and digestive disorders (14.10%). *Triticum aestivum* had the highest CSI, RFC and RPL levels at 8.00, 0.96, 1.00, respectively, followed by *Oryza sativa* and *Poa annua*. Likewise, *T*. *aestivum* and *Saccharum spontaneum* had 100% FL and ROP. Jaccard index ranged from 12.25 to 0.37. Twelve plant species namely *Chrysopogon zizanioides* (anti-inflammatory), *Pennisetum lanatum* (improve bull fertility), *Cymbopogon citratus* (glandular secretion), *Sorghum saccharatum* and *Themeda triandra* (malaria), *Aristida funiculate* (anticancer), *Koeleria argentia* (skin allergies), *Tetrapogon villosus* (antibacterial), *Cynodon radiatus* (eyes infection), *Sporobolus nervosa* (Jaundice), *Enneapogon persicus* (antifungal), and *Panicum repens* (dysfunctional cattle organs) were reported for the first time, with novel ethnoveterinary uses. The inhabitants of the study area had a strong association with their surrounding plant diversity and possessed significant knowledge on therapeutic uses of Poaceae to treat various health disorders in animals. Plant species with maximum cultural and medicinal values could be a potential source of novel drugs to cure health disorders in animals and human as well.

## Introduction

Botanical taxa belonging to the family Poaceae are the most substantial component of agricultural crops and livestock feed as well as the main sources of economy and revenue for many people of the rural areas around the globe [1]. The majority of livestock depends on forage grasses and natural pastures. Native plant species of Poaceae are a cost-effective source of nutrients for livestock and contribute significantly to conserve the soil integrity, water supply and air quality [2]. The major constraints for improved productivity of livestock is the low quality of forage available during the dry season, that cannot meet the nutrient requirements of grazing ruminants [3]. Rural populations worldwide use grasses as a source of feed for domestic animals and as medicines to treat health disorders in animals and humans [4].

Many scientific studies have recorded ethnoveterinary data on medicinal plants in different part of the world like, Kenya [5], Italy [6], Canada [7], South Africa [8], Pakistan [9], Brazil [10], Argentina [11], India [12], Nigeria [13], Spain [14] and Uganda [15], nevertheless Poaceae remained among the least explored plant family. In the past, grasses rich in nutrition were preferred over those that have therapeutically important compounds [4]. Grasses are of particular importance in traditional health care system due to the presence of biologically active compound like alkaloids, flavonoids and saponins [16]. The presence of alkaloids makes them highly resistant against foreign microbes while flavonoids have been shown to have anti-inflammatory, anticancer, and antiviral activity, and also help animals to repair oxidative cell damage [17]. *Cynodon dactylon*, *Sacchrum spontaneum*, and *Imperata cylindrica* have been found to be effective in inflammation and fungal diseases in animals [18]. Moreover, *Chloris barbata* is used as disinfectant [19], while *Heteropogon contortus* has anti-microbial, anti-cancer, anti-inflammatory properties, and it also increases milk production in livestock [20].

The local inhabitants of rural areas of Punjab widely use grasses for ethnoveterinary purposes. For example, *Cynodon dactylon*, *Eleusine indica*, *Bromus japonicus*, *Phragmites australis*, *Eragrostis minor* and *Desmostachya bipinnata* are reported to treat various stomach problems whereas *Sorghum bicolor*, *Brachiaria ramosa*, *Arundo donax*, *Chrysopogon zizanioides*, and *Panicum antidotale* are used to treat microbial infections in cattle [4]. Indigenous people with long history of livestock rearing may have developed precious information about the potential forage resources [21]. They prefer to use grasses as fodder because these are highly palatable than other form of fodders [22]. Their palatability also depends on animal choice and may also be linked with their seasonal availability, morphological and chemical nature of plant [23]. Animals generally prefer fresh foliage over dried because leaves of grasses are rich source of protein and cellulose, and have low lignin than forbs and shrubs [24]. Grasses beside the nutritional and healthcare services also reduce the grazing pressure on other palatable species and improve the productivity in livestock [25].

Nevertheless, different workers have been reported the traditional uses of plant species from different areas of Punjab [26–34], but a little is known about the therapeutic potential of grasses for the treatment of various diseases in livestock [4]. Consequently, the present study was conducted with the aim to document the ethnomedicinal uses of plant species of Poaceae family from Punjab province of Pakistan, traditionally used for the treatment of various health disorders in livestock. Another objective was to explore the cultural significance of species and their popularity among different tribes on the basis of their usage in animal healthcare.

## Materials and methods

### Ethics statement

The study was authorized by the Institutional Human and Animal Ethics Committee through No. HPR/HAEC/254/2019. Verbal informed consent was obtained from each informant before conducting the interview process.

### Study area

**Fig 1:**
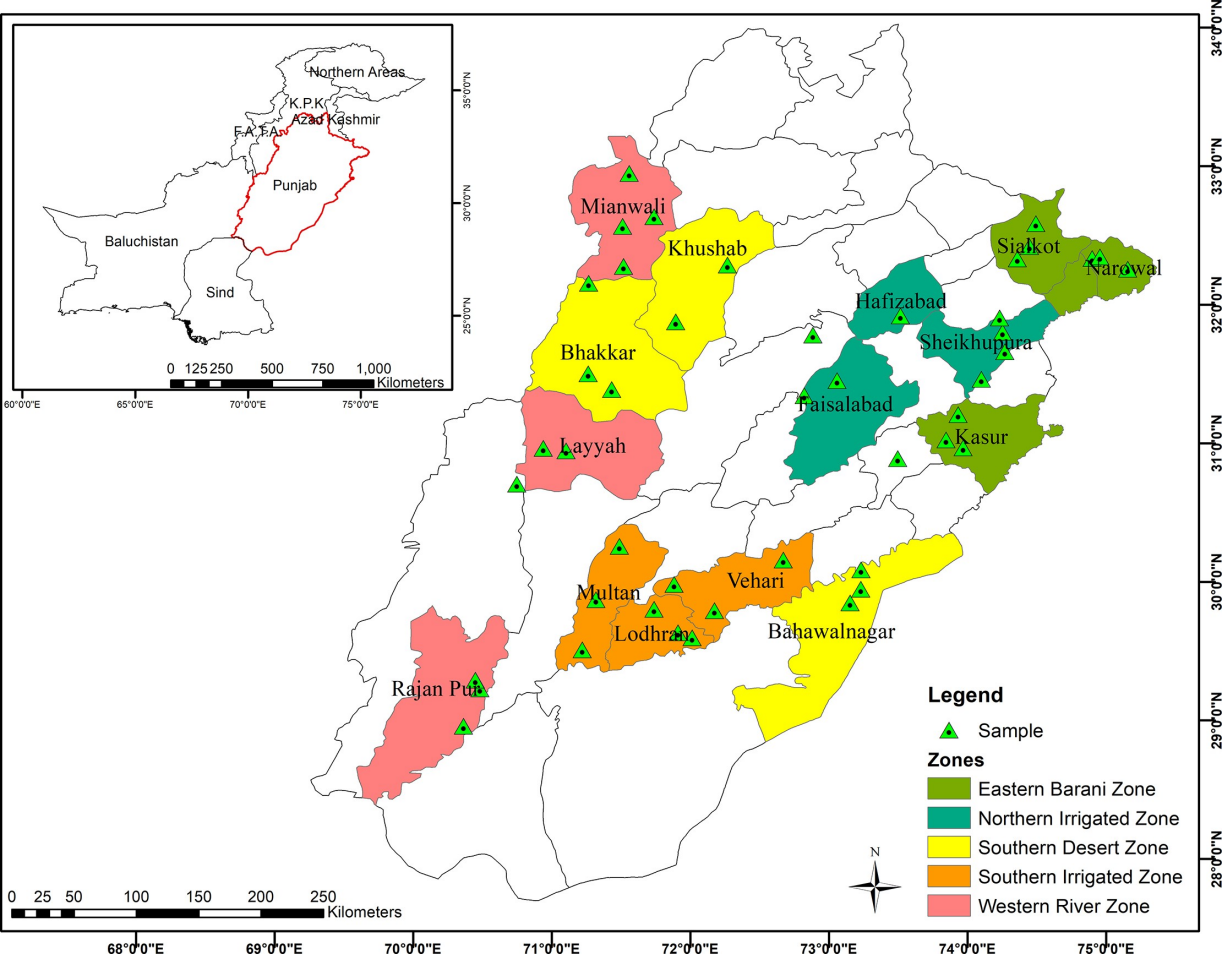
Map of study area showing different ecological zones and sampling sites of Punjab, Pakistan.

Punjab is the second largest province of Pakistan after Baluchistan. It encompasses of 205,344 km^2^ and is located between latitudes 27.42° and 34.02° N and longitudes 69.81° and 75.23° E at the northwestern edge of the geological Indian plate in South Asia [35]. Punjab is comprised of 36 districts and 5 agro-ecological zones [36] i.e. eastern barani zone, northern irrigated zone, southern desert zone, southern irrigated zone, and western river zone (Fig 1). Of these, northern irrigated zone and south desert zone are the two largest zones with large difference in cultural groups and ethnobotanical practices [4]. Most of the areas in Punjab consist of fertile alluvial plains heavily irrigated by 5 rivers namely Jehlum, Ravi, Chenab and Sutlej. Small deserts areas can be found in southern Punjab and Sulaiman Range. The variation in temperature and rainfall occurs all over the year. Soil of the study area is generally sandy, clay, and loamy [37]. Most of the areas experience foggy weather during winter and hot weather in summer while the average annual temperature ranges from -2°C to 45°C. June is the hottest and January is the coldest month of the year. Average annual rainfall of last five year is about 479.8 mm. The northern parts of the province receive a reasonable amount of rainfall through the year as compared to the drier southern part. Almost half of the rainfall occur during the month of July and August averaging about 255 mm. Punjab is home to over half of the total population of Pakistan. The ethnic composition of the area is quite diverse comprising of different tribes and communities. Rana, Gujjar, Butt, Rayain are the major ethnic groups. Most of the people speak Urdu and Punjabi languages followed by Saraiki. English language is used in government offices. Compared to the other provinces, Punjab has highest literacy rate. Moreover, Punjab contributes major share in the economy of Pakistan in terms of GDP. The economy of the people in the province is based on agriculture, and wheat is the widely cultivated crop with significant production of rice, cotton, corn, sugarcane, pulses and jute. The major occupation of the rural communities is farming, and they depend on agricultural means and livestock management to support livelihood. The inhabitants of the Punjab province have diverse traditional knowledge and practices because of linguistic and cultural variations. In agricultural lands like Punjab, grasses are preferred over other medicinal herbs and shrubs [4] because they are common, highly palatable and easy to process in order to cure livestock ailments [38].

### Data collection

Data on ethnomedicinal uses of grasses were collected through group discussions, semi-structured interviews and open and closed ended questionnaires during field visits in 2016–17, following the methods reported previously [39–41]. Prior to collect information, permission was obtained from the head of local government and prior informed consent from all local informants. In total, 271 participants including both men and women, traditional practitioners, village leaders, shepherds, cattle holders who worked in local farms and some senior household animal owners were interviewed. Demographic information (Table 1) about the participants was gathered by adopting a method of [42] and analysed in Microsoft Excel 365. Questionnaires were first developed in English, later translated in local languages, i.e. Punjabi, Saraiki and Urdu. Before conducting interviews, prior informed consent was also obtained from the participants after briefing the research objectives. No further ethical approval was required as there is lacking explicit rules or regulations pertaining to the practices of ethnomedicinal uses of plants or animals in Pakistan. However, participants were allowed to discontinue the interviews at any time.

**Table 1:** Demographic data about informants of the study area.

Variable	Demographic categories	Numbers	Percentage
Gender	Females	167	61.62
	Males	104	38.37
Age	Upto 20 years	31	11.43
	21–40 years	73	26.93
	41–60 years	119	43.91
	61–80 years	48	17.71
Occupation	Domestic cattle holders	121	44.64
	Nomads	80	29.52
	Farm cattle holders	70	25.83
Education levels	Illiterate	49	18.08
	Primary	77	28.41
	Middle	53	19.56
	Intermediate	37	13.65
	Graduate	28	10.33
	Master	16	5.90
	M.Phil.	9	3.32
	Ph.D.	2	0.74
Traditioinal practitioners			
	≤ 3	34	12.55
	3–5 years	66	24.35
	5–10 years	61	22.51
	10–15 years	55	20.30
	≥ 15 years	55	20.30

Collected plant specimens were identified with the help of the Flora of Pakistan, and names were verified through literature [43], www.efloras.org/index.aspx and Kew grass data base (https://www.kew.org/data/grasses-db/index.htm). For voucher specimen, standard herbarium techniques [44, 45] were strictly followed. All plants were labelled and deposited in the herbarium at the Department of Botany, University of Gujarat, Punjab, Pakistan and their voucher specimens were preserved for the furture record.

### Cultural significance index (CSI)

The relationship between use reports of a given species and agreement among the informant knowledge was attributed through cultural significance index (CSI). It was calculated following a method of [46] using formula:

CSI=Σ(i×e×c)×CF

Where *i* is the management of species having considerable impact on community (a species cultivated, managed or operated by any mean is awarded score of 2 and the value 1 is awarded if species is yet free from any kind of manipulation), *e* is the use preference of the informant for one plant species over another species for a specific purpose (value 2 is for preferred species and value 1 is for non-preferred species), *c* is use frequency of a plant species (value 2 is attributed to a high potential plant species being considerably used by informants and value 1 is awarded to a rarely cited species), correction factor (CF) is level of the informant consensus which comes from species citation divided by the number of citations of the most mentioned species.

### Relative frequency of citation (RFC)

RFC is to set up the priority order among the listed species and its value is depended on the numbers of participants who have mentioned a particular species as a medicinal plant or good fodder indicating its significance. The RFC was estimated with the help of the following equation following [47].

$$RFC=\frac{FC}{N}\;(0<RFC<1)$$

Where, FC is the number of participants who stated a particular plant species as an excellent medicinal plant and N is the total number of participants included in the study.

### Use value (UV)

Use value (UV) was calculated by applying a standard procedure reported previously [48, 49].

$$UV=\frac{U}{n}$$

Where U is the total number of use reports mentioned by informants for a given plant and n is the total number of informants interviewed for a given plant species. UV close to 1 indicates many use reports for a given plant and its importance among informants.

### Correlation study

Correlation between CSI, RFC and UVs was tested using Pearson’s correlation in SPSS 16.0 (SPSS for Windows, Chicago, IL, USA).

### Fidelity level (FL)

FL comes from the percentage of informant knowledge who report the uses of a plant species for an ailment and was determined using formula as previously reported by [48, 49].

$$FL(\%)=\times \frac{Np}{N}\times 100$$

Where, N*p* is the number of participants who reported the use of a grass for a specific purpose and N is the sum of participants who claimed the use of a grass for any purpose. High level of FL reflects the high use of plant species in specific disease in the study area.

### Relative popularity level (RPL)

Plant healing potential cannot be differentiated when species show same fidelity level. In order to differentiate the healing potential of species with same FL values, the relative popularity level is calculated, which is the ratio between ailments cured by a specific plant and total number of informants who reporting that disease. Base of RPL, plant species are divided into popular and non-popular groups. Popular species are those reported by more than half number of informants or above and rest of the species are declared as non-popular. For popular plant species RPL was arbitrarily selected equal to 1 that represents the complete popularity of a species for the cure of ailments and 0 value represents that no ailment was treated by this species [36].

### Rank order priority (ROP)

Plant species having different FL and RPL values were attributed with the correction factor (ROP) to rank properly the reported species. The ROP was calculated by multiplying FL and RPL values as elucidated earlier [47].

ROP=FL×RPL

### Jaccard index (JI)

The data presented in our study was compared with already published data in the adjacent areas of Himalayan territory using Jaccard index by appraising percentage of reported species and their medicinal uses [5].

$$JI=\frac{c\times 100}{a+b-c}$$

Where, a represent the number of plants in an area A, b is the number of plants in area B and c is the number of plants common to area A and B.

## Results and discussion

### Demographic features

**Fig 2:**
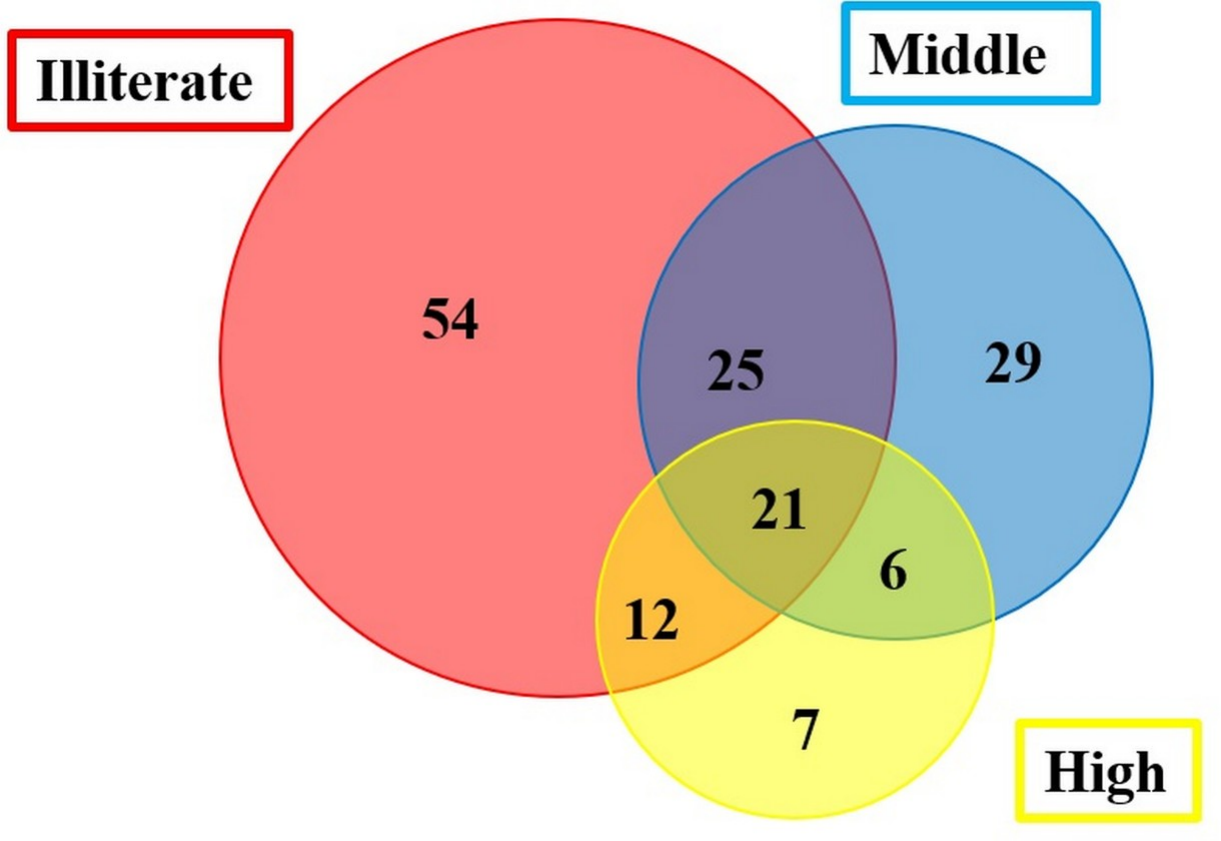
Venn diagram representing the overlap of taxa cited by different education groups. a) Illiterate, b) Middle education (primary to secondary), and c) Higher education (secondary to PhD).

**Fig 3:**
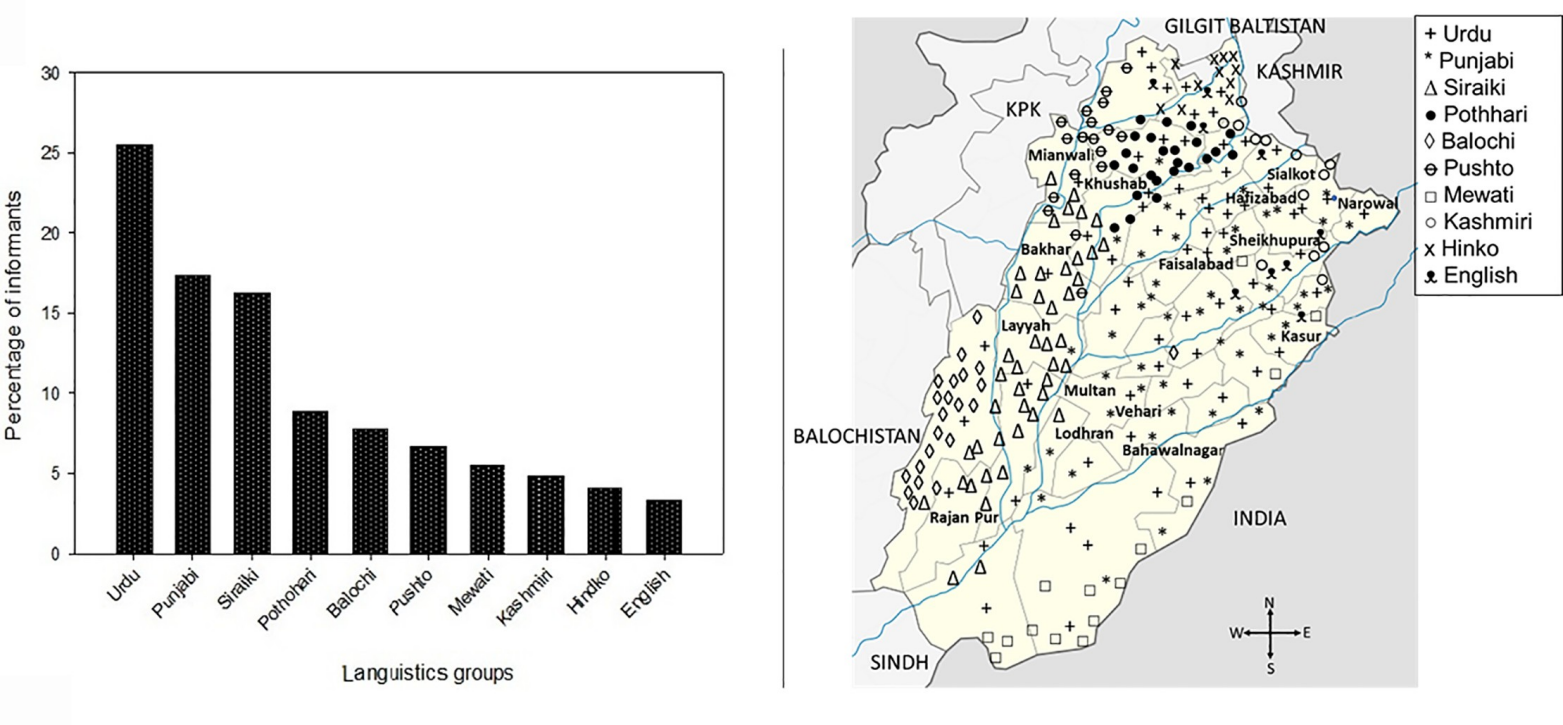
Linguistic wise classification and geographic distribution of the informants.

Data were collected from 271 informants (167 females and 104 males) of ages between 20 to 80 years (Table 1), including domestic cattle holders (44.64%), nomads (29.52%), and farm cattle holders (25.83%), and Traditional practitioners (TP) were classified into five groups based on their experience, such TP with less than 3 years of experience (34), 3–5 years (66). 5–10 years (61), 10–15 years (55), and more than 15 years (55). About, 18.08% informants were illiterate while others were educated up to master and PhD level. The plant citations were grouped in three education groups such as Illiterate, middle (primary to secondary), and high (secondary to PhD) and are represented through Venn diagram (Fig 2). The total plant species cited by each group were roughly equal to 112, 76 and 46 for the illiterate, middle and high education groups, respectively. About 54 species were only cited by illiterate people, 29 by middle, and 7 by highly educated people. Furthermore, there were common citations between different groups: 25 between illiterate and middle, 12 between illiterate and high, and 6 between middle and high. A total of 21 species were used by all three groups. From this analysis, it is easy to perceive that the level of indigenous knowledge on medicinal uses of the plant species of family Poaceae was more prevalent in illiterate participants and less educated people, while the more educated informants were less conversant on the ethnomedicinal uses of plant species, chiefly of Poaceae taxa. This difference in ethnomedicinal knowledge could be linked to the fact that educated people have highlevel of exposure to modernization and their dependence on allopathic medicines which have already been reported in literature [50, 51]. Most of the previous studies were focused on a single community with one ethic group and same culture [52], but in the last few decades, ethnobiologists have shown more interest in cross cultural variation of traditional knowledge of different communities and ethnic groups [53]. Because of this, we also collected data on ethno-veterinary uses of Poaceae from different ethnic groups i.e. Punjabi, Gujjar, Butt, Khawaja, Arayeen, and Rana. These groups have diverse culture and languages such as Urdu, Punjabi, Saraiki, Pothare, Balochi, Pashto, Mewatti, Kashmiri, Hindku, and English (25.46, 17.34, 16.24, 8.86, 7.75, 6.64, 5.54, 4.80, 4.06 and 3.32%, respectively (Fig 3).

### Taxonomic description of Poaceae species

In total, 149 species of Poaceae, belonging to 18 tribes were documented, which were used to treat various animal health disorders, classified into 12 major disease categories. Of these, 56% were of perennial nature and rest were annual herbs (S1 Table).

### Plant part (s) used

**Fig 4:**
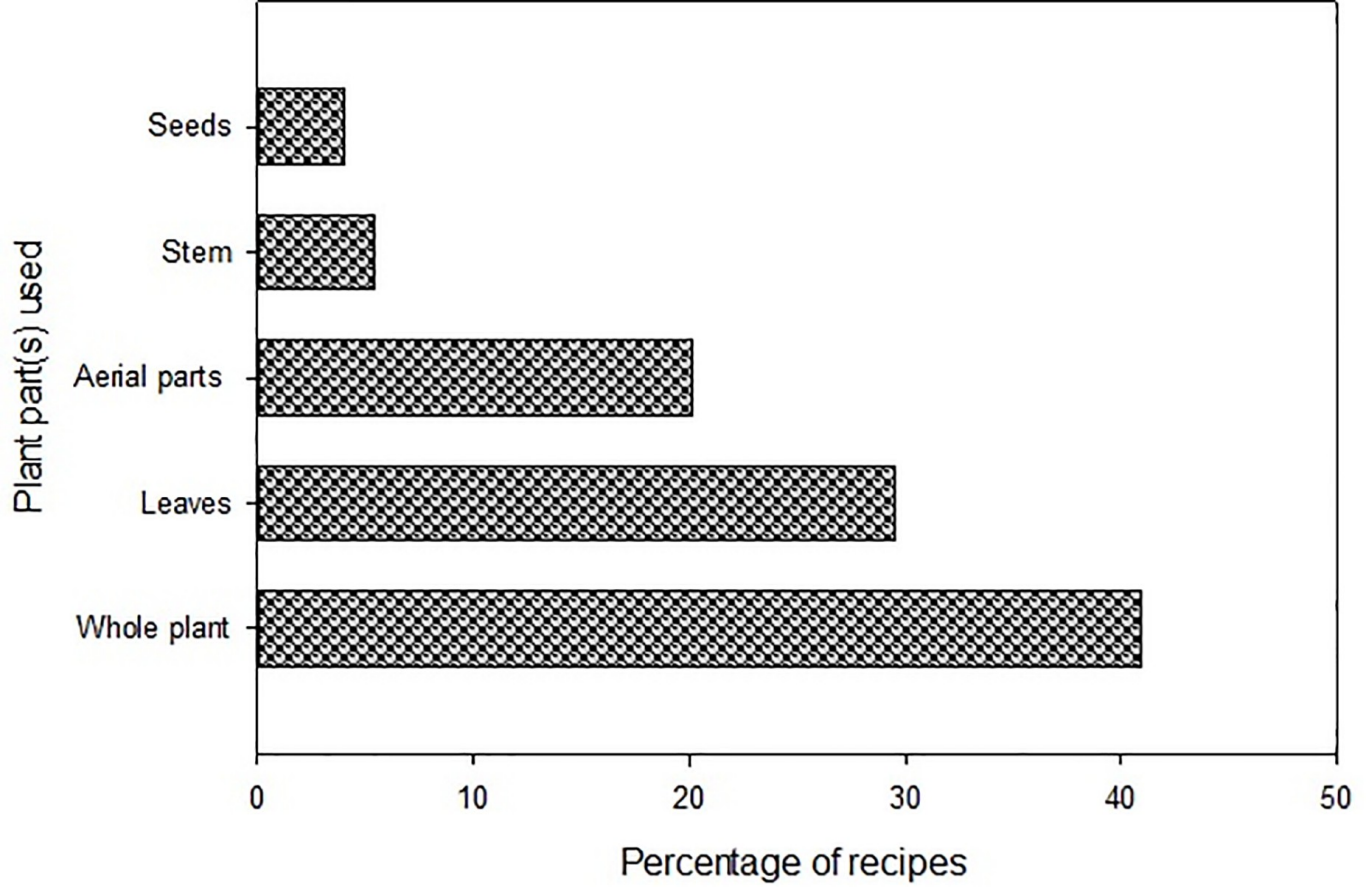
Proportion of plant part (s) used in different recipes for the disease management.

As depicted in Fig 4 and S1 Table, about 41% recipes were based on whole plants while leaves, aerial parts (without roots), stem, and seeds contributed to the remaing 59% of recipes. As the majority of the Poaceae taxa are small annual herbs with shallow roots, therefore, they are easy to pull out as a whole plant and utilized to treat various diseases [4]. Likewise, leaves are easy to collect and are rich in health beneficial secondary metabolites which contribute significantly to the treatment and prevention of health disorders [50, 54, 55]. Leaves have also been reported previously as one of the most consistently used plant part for grazing and medicinal purposes [26, 36, 56].

### Method of preparation and administration

**Fig 5:**
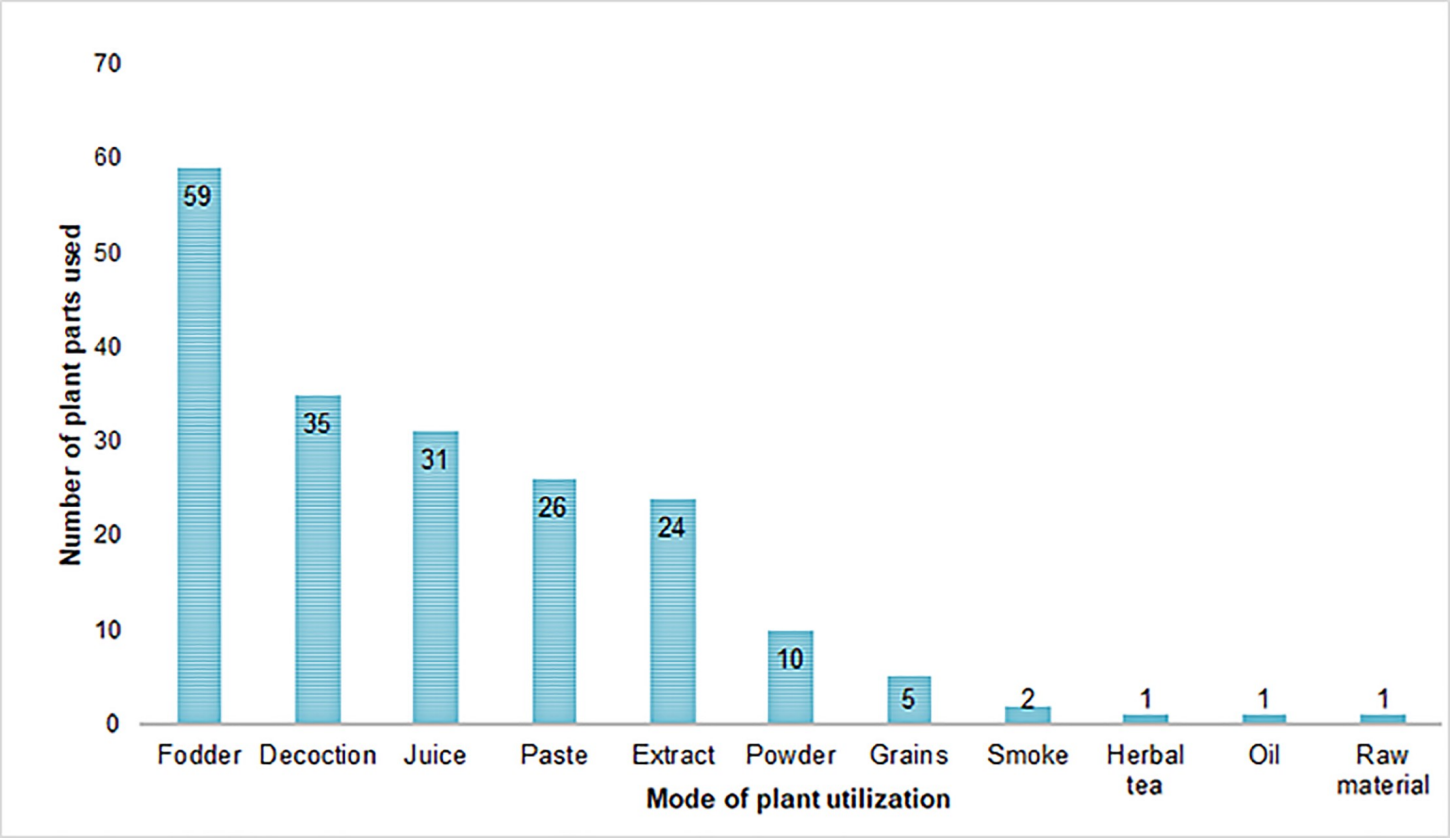
Commonly used methods in the preparation of herbal recipies.

As shown in Fig 5 and S1 Table, most of the plants were given orally as fodder (59 reports) without processing them in crude preparation followed by decoction (35 reports), juice (31 reports), paste (26 reports), extract (24 reports), powder (10 reports), grains (5 reports), smoke (2 reports) and herbal tea, oil, and processed raw material (1 report each). Offering plants as fodder to animals is the best way to treat a specific disease without having side effects. Crude preparation as decoction by boiling the plant parts in water for the treatment of various ailments is the most common practice among the ethnic communities in Punjab. Powder is prepared by grinding the dried plant parts and paste is made from crushing the fresh or dried plant parts with water or oil [34]. Futhermore, herbal preparations used to treat internal diseases i.e. gastrointestinal disorders, fever, and pain etc. were usually administrated orally, while for joint pain, skin infections topical method was used. Interestingly, there is a significant trend of multi-plant formulation devised by semiprofessional herbalists and traditional practitioners. In that case, powder of more than one plant/plant part is orally administered with water known as “Phakki”. There are certain cases with such recipes where overdose and malpractice resulted in adverse drug reactions.

### Major disease categories and use reportes

**Fig 6:**
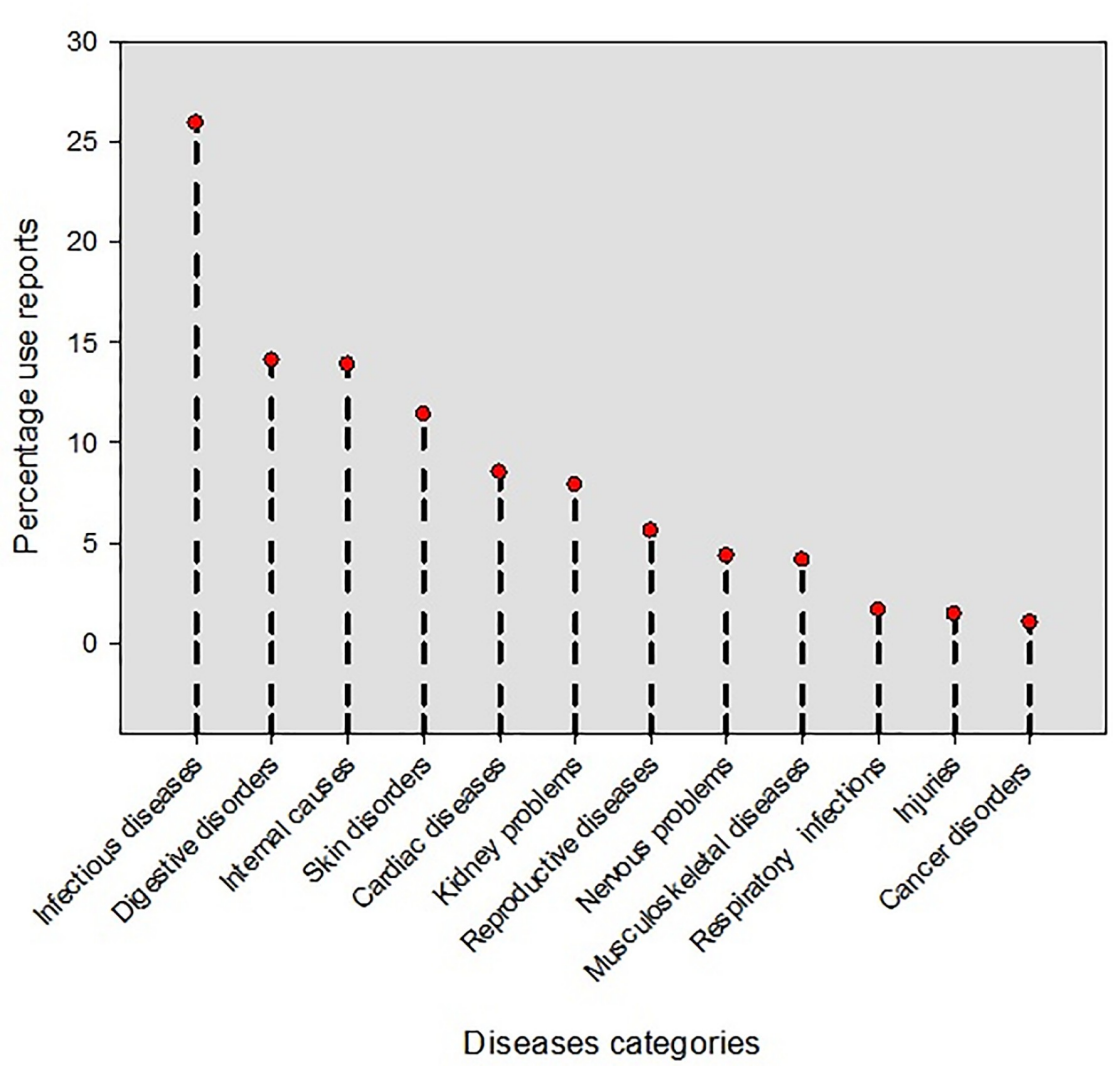
Major disease categories with use reports (%) in the study area. [Infectious diseases: cholera, tick infestation, typhoid fever, pneumonia, foot and mouth diseases, malaria, hemorrhagic fever, toothache, viral, fungal and bacterial infection, oral infections. Digestive disorders: stomachache, indigestion, gastro enteritis, abdominal pain, appetite, constipation, dysentery, diarrhea, dyspepsia. Internal causes: jaundice, toxification, piles, and general weakness. Skin disorders: allergies, herps, measles, soreness, shingles, itching. Cardiac diseases: blood pressure, heart palpitations, inflammation, anemia, diabetic. Kidney problems: urinary problems, urinary tract infections. Reproductive diseases: sexual disorder, premature ejaculation, menstrual discharge, dysfunctional organs, leucorrhoea, infertility. Nervous problems: hysteria, nervous exhaustion, headaches. Musculoskeletal diseases: bone fractures. Respiratory infections: bronchitis, cough. Injuries: skins injuries or cutting, wound healings. Cancer disorders: tumors.].

Inhabitants of the study area used 149 plant species to treat various health disorders in cattle, which were grouped into 12 major disease categories (S1 Table, Fig 6). The maximum number of use reports of plant species was reported for infectious diseases (25.93%), followed by digestive disorders (14.10%), internal causes (13.90%), skin diseases (11.41%), cardiac disorders (8.50%), kidney problems (7.88%), reproductive diseases (5.60%), nervous problems (4.35%), musculoskeletal diseases (4.14%), respiratory infections (1.65%), injuries (1.45%), and cancer (1.03%). Grasses mainly, *Eulaliopsis binata*, *Saccharum bengalense*, *Sorghum halepense*, *Phragmites australis*, *Paspalidium punctatum*, *Pennisetum divisum*, *P*. *lanatum Paspalum dilatatum*, *Setaria intermedia*, and *Brachiaria ramosa* were used to cure infectious diseases in livestock. Likewise, most of the skin disorders were treated with *Aristida adscensionis*, *Koeleria argentia*, *Cynodon dactylon*, *Acrachne racemosa*, *Panicum turgidum*, *Setaria glauca* and *Hordeum vulgare*.

In previous reports, *B*. *ramosa* and *S*. *bicolor* have been used in microbial infections [57], and *S*. *halepense* is primarily used for infectious diseases in livestock [58]. Futhermore, *D*. *bipinnata*, *E*. *indica* and *E*. *minor* are used to treat digestive disorders [4]. Likewise, *A*. *donax*, *C*. *dactylon* and *D*. *annulatum* are effective in gastrointestinal problems in animals [59–61]. Additionally, *E*. *indica* is use to relieve abdominal pain [62] and *C*. *citratus* is used as anti-bacterial, anti-fungal and anti-inflammatory agent [63]. We found *D*. *aegyptium* as detoxifier and antiallergic plant (S1 Table), but according to Kumar et al. [64], this plant has anti-inflammatory, anti-cancer, anti-microbial properties. Stem of *H*. *vulgare* is ground and mixed with water in order to increase the weight of cattle in Hawassa Zuria District, Sidama zone, Southern Ethiopia [65]. In Salt Range of Pakistan, flour made from *Hordeum vulgare* is used to cure jaundice [66, 67], *D*. *bipinnata* is diuretic and anti-amenorrhea [68], and root powder used to cure rheumatism while root infusion is used in urinary infections [69]. The grass *V*. *zizanoidesis* is antiseptic, demulcent and anti-inflammatory [70]. We reported that paste made from the leaves of *C*. *dactylon* is used to treat skin injuries in animals and juice is given for healing bone fractures. Additionally, root decoction is given to cattle having respiratory problem [67]. A decoction made from *S*. *bicolor* is given in in case of typhoid [71] whereas *Themeda anathera* is refrigerant and blood purifier [72]. The grass *D*. *bipinnata* is anti-amenorrhea and diuretic [73], while decoction made from the whole plant of *C*. *jwarancusa* is given in abdominal pain, tumor and unconsciousness [74]. We reported that *E*. *indica* is used in abdominal pain, which is in line with the study of Lans et al. [7]. Paste made from *A*. *mutica* is used to cure fungal diseases in livestock [75]. Zareen et al. [38] described that besides improving digestion, *B*. *bladhii* is also used as stored food and fodder for both livestock and wild ruminants. We found that extract made from aerial parts of *Z*. *mays* is used as detoxifier, nerve tonic and antiseptic while Zia-ud-Din et al. [57] reported that seeds of the same plant are mixed with oil to manage tick infection in livestock. We documented that the paste made from leaves of *I*. *cylindrica* is given to cattle in order to control microbial infections while Sushama and Nishteswar [76] reported that this plant is neuroprotective and possess anticancer properties. Juice extracted from leaves and roots of *S*. *spontaneum* improves appetite and gives relieve in inflammation, urinary problems, and abdominal pain [61]. *Dicanthium annulatum* is used in indigestion and menorrhagia [77].

During a detailed survey of literature review on ethnoveterinary practices, we have observed that ethno-veterinary literature on Poaceae used is fragmentary in Pakistan. To date, only few reports on ethno-veterinary investigations are available from different parts of Pakistan [9, 56, 61, 78–100]. On the other hand, rich data on ethnoveterinary practices is available from other parts of the world, for example, Africa, Orma land- Kenya [101], Nigeria [102], Zimbabwe [103], India [104], China [105], Netherlands [106], America [107], Canada [7] and Brazil [108]. Keep in view the current scenario, the current study is a continuance of earlier explorations for the improvement of records on the ethno-veterinary medication in Pakistan.

### Cultural significance index (CSI)

The cultural significance index is used to compute the importance of individual plant used by indigenous people. In CSI, the recognition or reputation of species is linked to its functions to the people and are considered auxiliary element in the cultural recognition of a plant [109, 110]. We observed that cultural importance of each species fluctuates between local communities. For instance, CSI values varied from 0.13 to 8.00, markedly affected by the preference, management and frequency of use by the local inhabitants (Table 2). This difference was influenced by the level of knowledge, the cultural settings, and the local conditions.

**Table 2:** Tribe wise distribution of plants of Poaceae with frequency of citations (FC), relative frequency of citations (RFC), use value (UV) and cultural significance index (CSI) values.

Tribe	S. #	Botanical name	FC (n)	RFC	UV	CSI
Andropogoneae	1.	*Apluda mutica* L.	47	0.17	0.56	0.18
	2.	*Bothriochloa bladhii* (Retz.) S.T. Blake	220	0.81	0.21	1.68
	3.	*Chrysopogon aucheri* (Boiss.) Stapf	70	0.26	0.47	0.27
	4.	*Chrysopogon serrulatus* Trin.	86	0.32	0.31	0.33
	5.	*Chrysopogon zizanioides* (L.) Roberty	111	0.41	0.12	0.84
	6.	*Cymbopogon citratus* (DC) Stapf	175	0.65	0.89	2.68
	7.	*Cymbopogon commutatus* (steud) Stapf.	88	0.32	0.74	0.34
	8.	*Cymbopogon jwarancusa* (Jones.) Schult	168	0.62	0.66	2.57
	9.	*Cymbopogon martini* (Roxb.) W. Watson	53	0.20	0.81	0.81
	10.	*Dichanthium annulatum* (Forssk.) Stapf	251	0.93	0.17	1.92
	11.	*Dichanthium foveolatum* (Delile) Roberty	98	0.36	0.19	1.5
	12.	*Eulaliopsis binata* (Retz) C.E.Hubb.	96	0.35	0.09	0.03
	13.	*Heteropogon contortus* (L.) P Beauv. ex. Roem & Schult	172	0.63	0.10	2.62
	14.	*Imperata cylindrica* (L.) Raeuschel	183	0.68	0.23	2.80
	15.	*Saccharum arundinaceum* Retz.	202	0.75	0.46	1.55
	16.	*Saccharum bengalense* Retz.	233	0.86	0.75	3.56
	17.	*Saccharum spontaneum* L	116	0.43	0.80	1.77
	18.	*Saccharum ravennae* L.	145	0.54	0.55	1.11
	19.	*Saccharum officinarum* L.	233	0.86	0.91	7.14
	20.	*Sorghum bicolor* (L.) Moench	223	0.82	0.91	3.56
	21.	*Sorghum saccharatum* (L.) Moench	178	0.66	0.64	0.68
	22.	*Sorghum halepense* (L.) Pers.	219	0.81	0.60	3.35
	23.	*Themeda anathera* (Nees) Hack	89	0.33	0.25	0.34
	24.	*Themeda triandra* Forsk.	134	0.49	0.15	0.51
	25.	*Vetiveria zizanioides* (L.) Nash	144	0.53	0.72	2.12
	26.	*Zea mays* L.	221	0.82	0.74	6.77
Aristideae	27.	*Aristida adscensionis* L.	143	0.53	0.34	1.15
	28.	*Aristida cyanantha* Nees ex Steud.	99	0.37	0.36	0.37
	29.	*Aristida funiculata* Trin. & Rupr.	55	0.20	0.29	0.21
	30.	*Aristida hystricula* Edgew,	156	0.58	0.20	0.59
	31.	*Aristida mutabilis* Trin. & Rupr.	51	0.19	0.14	0.25
	32.	*Stipagrostis plumosa* (L.) Munro ex T.Anderson	161	0.59	0.10	1.23
Arundineae	33.	*Arundo donax* L.	225	0.83	0.48	3.44
	34.	*Phragmites australis* (Cav.) Trin. ex Steud.	48	0.18	0.39	0.36
	35.	*Phragmites karka* (Retz.) Trin. ex Steud.	159	0.59	0.35	1.22
Aveneae	36.	*Avena fatua* L.	149	0.55	0.54	1.14
	37.	*Agrostis gigantea* Roth.	87	0.32	0.27	0.33
	38.	*Agrostis viridis* Gouan	107	0.39	0.71	0.41
	39.	*Avena sativa* L.	239	0.92	0.86	7.33
	40.	*Avena sterilis* (Dur.) Gill & Magne	39	0.14	0.28	0.15
	41.	*Koeleria argentina* Griseb.	79	0.29	0.63	0.30
	42.	*Phalaris minor* Retz.	176	0.65	0.22	1.34
	43.	*Polypogon monspeliensis* (L.) Desf.	97	0.36	0.49	0.37
	44.	*Trisetum clarkei* (Hook. f.) R. R. Stewart	55	0.20	0.33	0.21
	45.	*Polypogon fugax* Nees ex Steud	45	0.17	0.37	0.17
Bromeae	46.	*Bromus catharticus* Vahl	70	0.26	0.18	0.26
Bambuseae	47.	*Bambusa glaucescens* (Willd.) Merr.	183	0.68	0.56	0.70
Bromeae	48.	*Bromus japonicus* Thunb.	82	0.30	0.37	0.31
	49.	*Bromus pectinatus* Thunb.	67	0.25	0.17	0.25
	50.	*Bromus sericeus* Drobov	113	0.42	0.24	0.43
Chlorideae	51.	*Tetrapogon cenchriformis* (A. Rich.) Clayton	77	0.28	0.19	0.29
	52.	*Tetrapogon tenellus* (Roxb.) Chiov.	144	0.53	0.15	0.55
	53.	*Tetrapogon villosus* Desf..	43	0.16	0.21	0.16
Cynodonteae	54.	*Chloris gayana* Kunth	129	0.48	0.10	0.49
	55.	*Chloris barbata* Sw.	228	0.84	0.16	0.87
	56.	*Chloris dolichostachya* Lag.	137	0.51	0.02	0.52
	57.	*Chloris virgata* Sw.	211	0.78	0.26	1.62
	58.	*Cynodon dactylon* (L.) Pers.	249	0.92	0.60	7.34
	59.	*Cynodon radiaus* Roth.	105	0.39	0.38	0.40
Danthonieae	60.	*Schismus arabicus* Nees	216	0.80	0.24	0.83
Eragrostideae	61.	*Acrachne racemosa* (Heyne ex Roth) Ohwi	132	0.41	0.31	1.02
	62.	*Aeluropus lagopoides* (L.) Thwaites	71	0.26	0.33	0.27
	63.	*Dactyloctenium* aristatum Link.	46	0.17	0.30	0.17
	64.	*Dactyloctenium aegyptium* (L.) Wild.	178	0.66	0.30	2.73
	65.	*Dactyloctenium scindicum* Boiss.	98	0.36	0.25	0.75
	66.	*Desmostachya bipinnata* L. Stapf	196	0.72	0.41	1.50
	67.	*Eragrostis amabilis* (L.) Wight & Arn.	205	0.76	0.46	0.78
	68.	*Eragrostis atrovirens* (Desf.) Trin. ex. Steud.	81	0.30	0.34	0.31
	69.	*Eragrostis barrelieri* Dav.	127	0.47	0.24	0.48
	70.	*Eragrostis ciliaris* (L.) R. Br	224	0.86	0.15	0.85
	71.	*Eragrostis cilianensis* (All.) Janch.	148	0.55	0.14	0.56
	72.	*Eragrostis japonica* (Thunb.) Trin.	48	0.18	0.12	0.72
	73.	*Eragrostis pectinacea*. (Michx.) Nees	139	0.51	0.11	0.53
	74.	*Eragrostis minor* Host.	219	0.81	0.13	1.68
	75.	*Eragrostis pilosa* (L.) P. Beauve	52	0.19	0.22	0.21
	76.	*Eragrostis papposa* (Roem. & Schult.) Steud.	147	0.57	0.19	0.56
	77.	*Leptochloa panicea* (Retz.) Ohwi	39	0.14	0.21	0.31
	78.	*Leptochloa chinensis* (L.) Nees	67	0.25	0.32	0.25
	79.	*Eleusine indica* (L.) Gaertn	143	0.53	0.34	2.3
	80.	*Sporobolus virginicus* (L.) Kunth	46	0.17	0.35	0.17
Paniceae	81.	*Sporobolus nervosa* (Hocshsst.)	145	0.54	0.28	0.55
Eragrostideae	82.	*Dactyloctenium aristatum* Link	231	0.85	0.29	0.88
	83.	*Dactyloctenium scindicum* Boiss.	189	0.70	0.19	0.72
Hainardeae	84.	*Parapholis strigosa* (Dum.) C. E. Hubbard	43	0.16	0.13	0.16
Oryzeae	85.	*Oryza sativa* L.	255	0.94	0.76	7.81
Pappophoreae	86.	*Enneapogon shimpranus* (Hochst. ex A. Rich) Renvoize	69	0.25	0.36	0.264
	87.	*Enneapogon persicus* Boiss.	46	0.17	0.04	0.17
	88.	*Enneapogon desvauxii* P. Beauv.	56	0.21	0.37	0.21
Paniceae	89.	*Digitaria nodosa* Parl.	201	0.74	0.52	0.77
	90.	*Brachiaria distachya* (L.) Stapf.	151	0.56	0.19	1.14
	91.	*Brachiaria deflexa* (Schumach.) C.E.Hubb. ex Robyns	169	0.62	0.27	0.65
	92.	*Brachiaria mutica* (Forssk.) Stapf.	142	0.52	0.33	2.26
	93.	*Brachiaria eruciformis* (sm) Griseb.	159	0.59	0.26	0.61
	94.	*Brachiaria adspersa* (Trin.) Parodi	184	0.68	0.11	0.70
	95.	*Brachiaria ovalis* Stapf	148	0.55	0.09	0.56
	96.	*Cenchrus biflorus* Roxb.	163	0.60	0.16	1.25
	97.	*Cenchrus ciliaris* L.	238	0.69	0.46	1.82
	98.	*Cenchrus prieurii* (Kunth.) Marie	173	0.64	0.27	0.66
	99.	*Cenchrus pennisetiformis* Steud.	186	0.69	0.24	1.42
	100.	*Cenchrus setiger* Vahl.	135	0.50	0.08	0.52
	101.	*Digitaria pennata* (Hochst.) T.Cooke	52	0.19	0.07	0.21
	102.	*Digitaria ciliaris* (Retz.) Koeler	147	0.54	0.42	0.56
	103.	*Digitaria longiflora* (Retz.) Pers.	88	0.32	0.16	0.68
	104.	*Digitaria stricta* Rotch	221	0.82	0.25	0.85
	105.	*Digitaria radicosa* (Presl) Miq.	67	0.25	0.10	0.25
	106.	*Digitaria sanguinalis* (L.) scop.	173	0.64	0.58	0.66
	107.	*Digitaria setigera* Roth.	232	0.86	0.32	0.88
	108.	*Digitaria violascens* Link.	51	0.19	0.12	0.21
	109.	*Echinochloa colona* (L.) Link	182	0.67	0.21	1.38
	110.	*Echinochloa crus-galli* (L.) P. Beauv.	98	0.36	0.28	0.74
	111.	*Ochthochloa compressa* (Forssk.) Hilu.	152	0.56	0.33	0.58
	112.	*Panicum antidotale* Retz.	191	0.70	0.73	0.73
	113.	*Panicum atrosanguineum* Hochst. Ex A. Rich	43	0.26	0.57	0.16
	114.	*Panicum maximum* Jacq.	139	0.51	0.61	2.13
	115.	*Panicum turgidum* Forssk	81	0.30	0.68	0.31
	116.	*Panicum sumatrense* Roth.	104	0.38	0.62	0.39
	117.	*Paspalidium geminatum* (Forssk.) Stapf	93	0.34	0.44	0.35
	118.	*Paspalidium flavidum* (Retz.) A. Camus	86	0.32	0.47	0.33
	119.	*Paspalidium punctatum* (Burm.) A. Camus	41	0.15	0.24	0.15
	120.	*Paspalum paspaloides* (Michx.) Scribner.	84	0.31	0.08	0.32
	121.	*Panicum repens* L.	213	0.39	0.15	1.64
	122.	*Pennisetum divisum* (Forssk. ex J.F.Gmel.) Henrard	94	0.35	0.22	0.72
	123.	*Pennisetum glaucum* (L.) R.Br.	100	0.37	0.18	0.76
	124.	*Pennisetum americanum* (L.) Leeke	38	0.14	0.09	0.15
	125.	*Pennisetum orientale* Rich	211	0.78	0.55	0.81
	126.	*Sporobolus ioclados* (Nees. ex. Trin.) Nees.	90	0.33	0.75	0.34
	127.	*Paspalum dilatatum* Poir.	36	0.13	0.33	0.14
	128.	*Setaria glauca* (L.) P. Beauv	228	0.84	0.48	0.87
	129.	*Setaria intermedia* Roem. & Schult.	220	0.81	0.36	0.84
	130.	*Setaria italica* (L.) P.Beauv.	97	0.36	0.24	0.74
	131.	*Setaria pumila* (Poir) Roem. & Schult.	189	0.70	0.20	1.44
	132.	*Setaria verticillata* (L.) P. Beauv.	167	0.62	0.23	1.28
	133.	*Setaria viridis* (L.) P. Beauv.	241	0.89	0.16	0.92
	134.	*Brachiaria prostrata* (Lam) Griseb	231	0.85	0.33	0.88
	135.	*Brachiaria ramosa* (L.) Stapf	201	0.74	0.07	0.77
	136.	*Pennisetum lansatum* Klotzsch.	160	0.59	0.06	0.61
	137.	*Urochloa panicoides* P. Beauv.	143	0.53	0.11	0.55
	138.	*Urochloa setigera* (Retz.) Stapf.	233	0.86	0.09	0.89
Poeae	139.	*Dactylis glomerata* L.	101	0.37	0.09	0.38
	140.	*Lolium temulentum* L.	35	0.13	0.68	0.13
	141.	*Lolium persicum* Boiss. & Hohen.	119	0.44	0.39	0.45
	142.	*Poa annua* L.	251	0.93	0.86	7.69
	143.	*Poa infirma* Kunth	249	0.92	0.72	0.95
Triticeae	144.	*Hordeum vulgare* L.	107	0.39	0.83	0.82
	145.	*Triticum aestivum* L.	261	0.96	0.93	8.00
Zoysieae	146.	*Leptothrium senegalense* (Kunth) Clayton	154	0.57	0.25	0.59
	147.	*Tragus berteronianus* Schult.	65	0.24	0.07	0.25
	148.	*Tragus racemosus* (L.) All.	54	0.20	0.06	0.21
	149.	*Tragus roxburghii* Panigrahi	83	0.31	0.04	0.32

The highest CSI value was found for *T*. *aestivum* (8.00), which is extensively used in anti-cancer and gastrointestinal disorders in livestock. The other 6 species with CSI were *O*. *sativa* (0.81), *P*. *annua* (7.69), *C*. *dactylon* (7.34), *A*.*sativa* (7.33), *S*. *officinarum* (7.14) and *Z*. *mays* (6.77). These species were highly cited and preferentially used by the informants to be used in therapeutic and other purposes but also cultivated and used different kind of management tools. The preference and frequency of use and quality of medicinal use are the factors that determine the cultural importance of plants. The high CSI values of plant species also reflect the fact that more a species is available to the users of a community for medicinal purpose, it becomes more important. For instance, *T*. *aestivum* is well known herb which is mentioned in Ayurveda herbal system. It has been used in many dietary supplements and is extremely valuable for treating various ailments such as kidney malfunctioning, immune modulator, joint swelling and bacterial infection [111]. Furthermore, species reported with high cultural significance are not only important for ethnic groups of Punjab but also for the other ethnic group of the world. Therefore, these species have accumulated a lot of traditional knowledge that has been transmitted through direct experience over the time in next generations. Grasses represented with lowest CSI values were, *L*. *temulentum* (0.13), *P*. *dilatatum* (0.14), *P*. *typhoidum* (0.15), *P*. *punctatum* (0.15), *A*. *sterilis* (0.15), *P*. *atrosanguineum* (0.16), *P*. *strigosa* (0.16), *T*. *villosus* (0.16), *E*. *persicus* (0.17), *S*. *arabicus* (0.17), *D*. aristatum (0.17), *P*. *fugax* (0.17), and *A*. *mutica* (0.18). The results obtained in our study are in agreement with the study of Wong [112], who observed that plants which are not easily available are less popular in the community, hence have lower CSI value. Nevertheless, Albuquerque et al. [113] proposed a weak correlation between use and availability of plants and further suggested that species with greater cultural significance tend to become vulnerable or rare locally.

### Relative frequency of citations (RFC) and use value (UV)

Relative frequency of citations reveals the importance of each grass among indigenous communities of Punjab, ethno-veterinary medicines and primary health care of animals to make them healthy and productive. It is calculated from the citation frequency of informants claiming the use of a plant species divided by the total number of informants who participated in the survey to share their indigenous knowledge [5]. In our work, RFC ranges 0.96 to 0.14 (Table 2).

The maximum RFC value was obtained for *T*. *aestivum* (0.96). The other high citation species and their respective values were *O*. *sativa* (0.94), *P*. *annua* (0.92), *D*. *annulatum* (0.92), *C*. *dactylon* (0.91), *P*. *infirma* (0.91) and *A*. *sativa* (0.91), *S*. *viridis* (0.88), *S*. *officinarum* (0.85), *S*. *bengalense* (0.85), *U*. *setigera* (0.85), *D*. *setigera* (0.85) and *B*. *prostrata* (0.85), *C*. *barbata* (0.84), *S*. *glauca* (0.84), *S*. *bicolor* (0.82), *Z*. *mays* (0.81), *S*. *intermedia* (0.81), *S*. *halepense* (0.80), and *E*. *minor* (0.80). These resutls highlight the fact that grasses are known to local culture for a long period of time and majority of the people in different regions of the Punjab were not fully aware of the medicinal potential of Poaceae due to thier poor educational background.

Relative frequency of citation highlights the importance of individual species among local communities based on the number of uses [114, 115]. It has been suggested that plants with high RFC values should be involved in biological, phytochemical and pharmacological studies for further investigation of drug development [56]. Furthermore, such plants must be conserved on priority basis due to the threat of over exploitation and extensive use by the community [19]. Values of RFC are very dynamic as it changes with area to area and depends on the folk knowledge of the native people. It is well known that species with low RFC values are not necessarily less important [116]. Their low values may represent the low knowledge of the local people especially the younger ones, who are not aware of the uses of these species.

We observed that plants with high RFC values depict their dominancy in the study area and indigenous people are more familiar with them. They prefer these plants over others because of their availability and positive role in traditional health system. These results are in line with the appearance hypothesis which explains that local people have greater knowledge of ethnomedicinal use on plants which are more common in an area [58]. Additionally, common plants would allow local people to gain more experience of their properties and consequently would have a greater probability of being introduced into the local culture [56].

Use value is an important index to identify the plant species which are extensively used among indigenous communities [117]. In our case, use values ranged from 0.02 to 0.93 (Table 2) and species with high UV were, *T*. *aestivum* (0.93), *S*. *bicolor* (0.91), *C*. *citratus* (0.89), *A*. *sativa* (0.86) *P*. *annua* (0.86), *H*. *vulgare* (0.83), and *C*. *martini* (0.81), and *S*. *spontaneum* (0.80). The high use values of species show their importance in the traditional medicine system [118], which can be attributed with the fact that they are the first choice of the traditional healers for the treatment of ailments and local inhabitants are familiar with these plants [58, 119]. Species with low use values were, *C*. *dolicostachya* (0.02), *E*. *persicus* (0.04), *P*. *repens* (0.05), *P*. *lanatum* (0.06) and *T*. *racemosus* (0.06). The low use value of plants may relect less distribution of species in the area and low ethnomedicinal knowledge of informants [58, 120]. On the other hand, high use citations of *T*. *aestivum* could be due to its prevelance, high nutritional values, and cheap source of forage with the additional benefit of potential medicinal properties.

### Correlation among ethnobotanical indices

**Fig 7:**
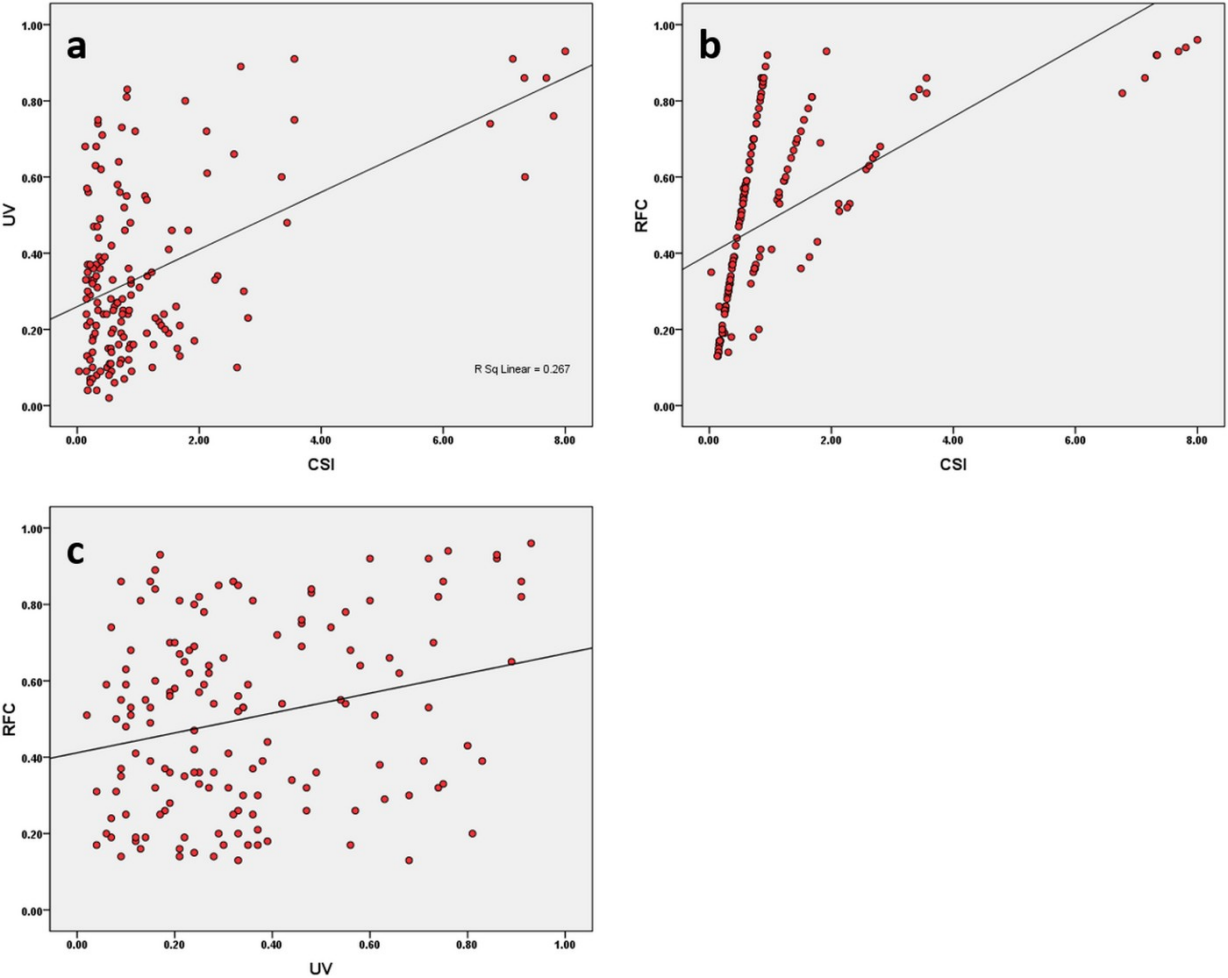
The scatter plot representing correlation. a) UV vs CSI, b) RFC vs SCI, and c) UV vs RFC.

The relationship among ethnobotanical indices i.e. CSI, RFC and UV were found significant at the 0.01 level (Fig 7, S2 Table). A positive correlation was present between CSI and RFC (r = 0.59**), followed by CSI and UV (r = 0.51**) and RFC and UV (r = 0.25**). The positive correlation provides evidence of cultural importance of each species and relative importance of the use of plants. The fact that these indices are strongly correlated means that their patterns across species match.

### Fidelity level (FL)

The fidelity level of 24 most important species ranged from 14.3 to 100% (Table 3). *Triticum aestivum* and *Saccharum spontaneum* shows 100% fidelity level for tumor and urinary problems. Other species with high FL values were: *C*. *jwarancusa* (typhoid fever), *V*. *zizanioides* (stomach pain), *S*. *officinarum* (digestive disorders), *T*. *tenellus* (antimicrobial), *D*. *bipinnata* (digestive problems), *S*. *bengalense* (oral infections), *E*. *minor* (anti-inflammatory), *O*. *sativa* (wound healing), *D*. *aegyptium* (jaundice), *P*. *minor* (cough) and *C*. *virgata* (bone fracture) with FL 98, 94, 84, 80, 78, 76, 75, 74, 71, 71 and 70%, respectively. The high fidelity score of a species highlights the existence of a disease in the study area and utilization of plant species by the local people in order to cure it [94, 95]. Plants are important and cheap source of medicine to treat multiple health issues in animals [121]. The traditional ethno-veterinary system has played a significant role in animal production especially in the rural areas where livestock diseases are locally treated [122, 123].

**Table 3:** Highly utilized plant species with FL, RPL and ROP.

Species name	I_u_	NA	Major ailments	I_p_	FL	RPL	ROP
*Triticum aestivum*	84	3	Anticancer	84	100	1	100
*Saccharum officinarum*	90	3	Digestive disorders	76	84	1	84
*Sorghum halepense*	80	4	Infectious diseases	55	69	1	69
*Cymbopogon jwarancusa*	84	4	Typhoid fever	82	98	1	98
*Saccharum bengalense*	71	7	Oral disorders	54	76	1	76
*Saccharum spontaneum*	71	4	Urinary pain	71	100	1	100
*Oryza sativa*	68	3	Diarrhea	50	74	1	74
*Vetiveria zizanioides*	65	3	Stomach pain	61	94	1	94
*Arundo donax*	62	5	Blood pressure	37	60	0.98	58
*Bambusa glaucescens*	57	4	Allergies	30	53	0.92	48
*Phragmites karka*	53	2	Nervous problems	25	47	0.86	41
*Imperata cylindrica*	51	6	Piles	25	49	0.86	42
*Cynodon dactylon*	42	8	Anemia	24	57	0.79	45
*Setaria pumila*	34	2	Oral infection	21	62	0.74	46
*Bromus japonicus*	29	2	Constipation	17	59	0.74	43
*Sporobolus ioclados*	29	5	Liver disorder	14	48	0.66	32
*Octhochloa compressa*	21	4	Kidney pain	14	67	0.58	39
*Panicum antidotale*	19	2	Antibacterial	10	53	0.53	28
*Phalaris minor*	14	1	Cough	10	71	0.5	36
*Dactyloctenium aegyptium*	14	4	Jaundice	10	71	0.45	32
*Tetrapogon tenellus*	10	4	Antimicrobial	8	80	0.45	36
*Chloris virgata*	10	2	Bon fracture	7	70	0.41	29
*Desmostachya bipinnata*	9	4	Digestive problems	7	78	0.36	28
*Eragrostis minor*	8	2	Anti-inflammatory	6	75	0.29	22

*Iu*: Sum of participants who claimed the use of a grass for any purpose *NA*: Number of ailments treated *Ip*: Number of participants who reported the use of a grass for specific purpose *FL*: Fidelity level *RPL*: Relative popularity level *ROP*: Rank order priority.

### Relative popularity of species

One hundred and forty-nine species were mentioned for different kind of diseases by 271 informants, interviewed during this study. Of these, 125 species were reported by fewer than 8 informants and therefore were excluded for further discussion. The rest of the 24 species were reported by more than 7 informants (Table 3). For species cited by 8 to 62 informants, the number of uses per species increased progressively (S1 Fig) with increasing number of informants interviewed showing positive correlation (r, 0.149). Conversely, species mentioned by more than 65 informants, the average number of uses per species did not increase with increasing number of informants. About sixteen plant species mentioned by 62 informants were grouped as unpopular whereas, eight species reported by 65 or more informants were classified as popular.

Species with high popularity level (1.0 RPL) were: *T*. *aestivum*, *S*. *officinarum*, *S*. *halepense*; *C*. *jwarancusa*, *S*. *bengalense*, *S*. *spontaneum*, *O*. *sativa* and *V*. *zizanioides*. The healing potential of each species may vary and is expressed by its FL value [36]. Rank order priority index can be used as correction factor to rank plants properly with different fidelity level [48, 49]. Out of 24 species only 9 attained 50% or above ROP values. This can be attributed to the decreasing popularity of herbal medicines in the study area. *T*. *aestivum* and *S*. *spontaneum* were reported with highest ROP value (100%), trailed by *C*. *jwarancusa* (98%), *V*. *zizanioides* (94%), *S*. *officinarum* (84%), *S*. *bengalense* (76%), *O*.*sativa* (74%), *S*. *halepense* (69%) and *A*. *donax* (58%). The rest of the species presented less than 50% of ROP values. The high popularity of these species can be attributed to their high nutritional values [4] and may be linked to the fact that local farmers are aware of these species and they frequently used them for the treatment of various ailments in the livestock. This is an agreement with similar findings of previously reported studies conducted in the same province, Punjab [4, 36]. Our study is also consistent with the findings of on the status of healing potential of medicinal plants in Palestinian area [48, 49] and medicinal plants among Bedouins communities in Negev desert [46].

### Jaccard index (JI)

The Jaccard Index was performed in order to develop a relationship between this study and previously reported studies, one from other regions of Pakistan (within Pakistan) and other from outside the Pakistan. This helped in finding a novelty in uses because ethnobotanical information may vary with respect to cultural differences and ethnic origin [53, 55]. Therefore, a comprehensive research with high understanding of ethnobotanical folk knowledge is mandatory for better judgement [124], and exploration of traditional knowledge in order to find novelty in work and possible drug discovery [99, 125].

### Relationship with studies from Pakistan

In comparison of our study with other studies form Pakistan, the JI ranged from 12.25 to 0.37 (Table 4). The highest JI (12.25) was found with previous report from Central Punjab [4], followed by the study conducted in Layyah, Punjab, Pakistan [125] with 5.61 JI, Swat, KPK, Pakistan [58] with 3.70 JI, Gujrat, Pakistan [126] with 3.63 JI and Hafizabad Punjab, Pakistan [36] with 3.63 JI. The lowest JI such as 0.37, 0.46, and 0.47 was recorded from Abbatobad, KPK, Pakistan [21], Hungu, Pakistan [73], and Mohmand Agency, FATA, Pakistan [51], respectively. Our study shares the greatest number of common species (53) with the study from Central Punjab, Pakistan [4] with 71.7% similar uses and 28.3% dissimilar uses (Table 4). Similarly, 11 species are common between our study and a study form Layyah, Punjab, Pakistan that reported a total of 78 species [125] with 7 species having similar uses and 5 species having dissimilar uses. 8 species, in our study, were found common to the studies reported from Gujrat, Punjab, Pakistan [126], Hafizabad, Punjab, Pakistan [36], and Swat, KPK, Pakistan [58] where a total of 88, 85 and 83 species, respectively, were recorded. Other studies from Pakistan only share 1 to 4 species common to our study.

**Table 4:** Comparison between this study and other studies from Pakistan. Evidences based comp.

Study area	Journal name	Ref.	TRS	CPBA	PPAA	PPSA	PSU	PDU	% SU	% DU	JI
District Attock, Punjab, Paksitan	Pak. J. Bot.	[27]	43	1	42	148	0	1	0.00	2.33	0.52
Talagang, Punjab, Pakistan	Braz. J. Pharmacol.	[127]	101	3	98	146	1	2	0.99	1.98	1.24
Gujrat, Punjab, Pakistan	Ethnobotanical Leaflets	[126]	88	8	80	141	7	1	7.95	1.14	3.63
Hafizabad, Punjab, Pakistan	Plosone	[36]	85	8	87	141	6	2	7.06	2.35	3.63
Lakki Marwat KPK, Pakistan	J. Ethnopharmacol.	[118]	62	3	59	146	1	2	1.61	3.23	1.48
Central Punjab, Pakistan	J. Ethnobiol Ethnomed	[4]	53	53	0	96	38	15	71.70	28.30	12.25
Layyah, Punjab, Pakisstan	Ind. Res. J. Pharm. Sci.	[125]	78	11	69	138	7	4	8.97	5.13	5.61
Toba Tek Singh, Punjab, Pakistan	Ind. Res. J. Pharm. Sci.	[119]	17	4	13	145	0	4	0.00	23.53	2.59
Abbottabad, KPK, Pakistan	J. Ethnopharmacol.	[73]	120	1	119	148	0	1	0.00	0.83	0.37
Sawat, KPK, Pakistan	Pak. J. Bot.	[58]	83	8	75	141	5	3	6.02	3.61	3.70
Karak, KPK, Pakistan	J. Ethnopharmacol.	[128]	46	3	43	146	3	0	6.52	0.00	1.61
Hangu, KPK, Pakistan	Evid Based Comp. Alt. Med.	[129]	67	1	66	148	0	1	0.00	1.49	0.46
Tehsil Kabal, KPK, Pakistan	J. Bot	[130]	138	3	135	146	1	2	0.72	1.45	1.07
Bajaur Agency, FATA, Pakistan	J. Ethnobiol Ethnomed.	[51]	64	1	63	148	1	0	1.56	0.00	0.47

Ref. References, TRS: Total reported species, CPBA: Common plants of both areas, PPAA: Plants only present in the aligned area. PPSA: Plants only present in the study area, PSU: Plants with similar uses, PDU: Plants with different uses

### Relationship with studies from outside the Pakistan

From outside Pakistan, the JI ranged from 0.49 to 1.99 (Table 5). The higher JI such as 1.99, 1.97 and 1.73 was found with the studies conducted in Ogun State, Nigeria [71], Terai Forest, Western Nepal [131], and tropical regions of Nigeria [132], respectively. The lower JI such as 0.49, 0.50, 0.50 and 0.60 was found with the studies reported from Eastern Amazon, Brazil [133], Tamil Nadu India [134], Baitadi & Darchula, Nepal [135], and Bandarban, Bangladesh [55], respectively. In contrast to Pakistan, our study shares only few common species with the studies from outside the Pakistan (Table 5). [71] reported 63 species from Ogun State, Nigeria and only 4 species are common to our study with 1.95% similar uses. Similarly, 4 species are found common to the studies conducted in Tropical regions of Nigeria by [132] and Guimaras Island, Philippines by [13] who have reported 93 and 142 species with 3.23 and 0.70% similar uses, respectively. overall, the percentage similar uses ranged from 0 to 4.55% and percentage dissimilar uses ranged from 0 to 4.76%.

**Table 5:** Comparison between this study and other studies from outside the Pakistan. Evidences based comp.

Study area	Journal name	Ref.	TRS	CPBA	PPAA	PPSA	PSU	PDU	% SU	% DU	JI
Tamil Nadu, India	Braz. J. Pharmacol.	[135]	54	1	53	148	1	0	1.85	0.00	0.50
Western Himalaya, India	J. Ethnobiol Ethnomed.	[136]	78	2	76	147	1	1	1.28	1.28	0.90
Bandarban, Bangladesh	Front. Pharmacol.	[55]	159	1	9	147	0	1	0.00	0.63	0.64
Baitadi & Darchula, Nepal	J. Ethnobiol Ethnomed.	[134]	53	1	52	148	0	1	0.00	1.89	0.50
Terai Forest, Western Nepal	J. Ethnobiol. Ethnomed.	[131]	66	2	62	145	2	0	3.03	0.00	1.97
Ogun State, Nigeria	Amer. J. Plant Sci.	[71]	63	4	59	145	1	3	1.59	4.76	1.99
Tropical regions of Nigeria	J. Ethnopharmacol.	[132]	93	4	89	145	3	1	3.23	1.08	1.73
Guimaras Island, Philippines	J. Ethnopharmacol.	[137]	142	4	138	145	1	3	0.70	2.11	1.45
Switzerland	J. Ethnobiol Ethnomed.	[138]	22	1	21	148	1	0	4.55	0.00	0.60
Uige, Northern Angola	J. Ethnobiol Ethnomed.	[139]	122	3	118	146	0	3	0.00	2.46	1.14
Eastern Amazon, Brazil	J. Ethnopharmacol.	[131]	56	1	55	148	0	1	0.00	1.79	0.49

Ref. References, TRS: Total reported species, CPBA: Common plants of both areas, PPAA: Plants only present in the aligned area. PPSA: Plants only present in the study area, PSU: Plants with similar uses, PDU: Plants with different uses

In general, the high degree of similar type of plant uses may indicate same cultural practices among communities, and similar type of vegetation may reflect same type of floral diversity and climate in those areas [41]. It has been reported that neighboring indigenous communities share more common traditional practice of plants as medicines in order to cure various ailments and this is because of more social trade and sharing of ethnomedicinal knowledge among native groups [140, 141]. In contrast, a low similarity index indicates less sharing of medicinal knowledge and low social interaction that could have been happened in the past bringing more difference in ethnobotanical practices [51]. Geological isolation of ethnic groups and plants resulted a significant change in vegetation structure and therapeutic uses of indigenous plants and this may be a reason for loss of ethnobotanical information [44]. A low degree of similarity index of our study with other studies conducted in same province or in other parts of Pakistan indicated that either a little attention has been paid towards grasses in these studies and zero (0) percent similarity index show that grasses were totally ignored. Therefore, this study represents the first comprehensive report on ethno-veterinary knowledge of indigenous grasses of the whole province of Punjab.

### Novelty in ethno-veterinary uses

Therapeutic uses of plant species of family Poaceae were compared with previous reports from different parts of the Pakistan and with other studies conducted in the neighboring areas including India, Bangladesh and Nepal to find out the novelty index. The data shows significant differences in the usage of plant species and their parts used to treat health disorders in animals. Out of 149 species, ethnomedicinal uses of 12 plant species such as *C*. *zizanioides* (anti-inflammatory), *P*. *lanatum* (improve bull fertility), *C*. *citratus* (glandular secretion), *S*. *saccharatum* and *T*. *triandra* (malaria), *A*. *funiculate* (anticancer), *K*. *argentia* (skin allergies), *T*. *villosus* (antibacterial), *C*. *radiates* (eyes infection), *S*. *nervosa* (Jaundice), *E*. *persicus* (antifungal), and *P*. *repens* (dysfunctional cattle organs) have rarely been reported so far from this region and rest of the world. It is noteworthy that most of these plants have not been explored pharmacologically. Therefore, they could be used for in-depth phytochemical screening and bioactivity assays in order to validate their traditional uses. Moreover, less familiar *P*. *annua*, *D*. *annulatum*, *P*. *infirma*, *S*. *viridis*, *S*. *bengalense*, *U*. *setigera*, *D*. *setigera*, *B*. *prostrata*, *C*. *barbata*, *S*. *glauca*, *S*. *intermedia* and *E*. *minor* with high citations should also be investigated in detail.

## Conclusion

The present study is the first comprehensive report with emphasis on the use of Poaceae in traditional ethno-veterinary medicine. A total of 149 species with therapeutic uses were reported, which represents new bioresources for pharmacological studies and drug discovery. Our study revealed that ethnobotanical knowledge of Poaceae is less prevailing in the rural areas of Punjab compared to other medicinal plants. Therefore, ethnomedicinal applications of the members of this family should be reported from other parts of the country, which would be helpful in conserving precious medicinal plant knowledge and its application in novel drug discovery.

## Supporting information

S1 Table Traditional ethnoveterinary uses of plants reported by the indigenous communities and local herbal practitioners of Punjab province. (DOCX)

S2 Table Correlation analysis between a) UV vs CSI, b) RFC vs SCI, and c) UV vs RFC.
(DOC)

S1 Fig Correlation scatter plot between number of informants and ailments. (TIF)
