# Supplementary material for: Ethno-veterinary uses of Poaceae in Punjab, Pakistan
Source: PLoS One. 2020 Nov 3;15(11):e0241705. doi: 10.1371/journal.pone.0241705 (PMC7608896; doi:10.1371/journal.pone.0241705)
Supplement: S1 Table — (DOCX) [file pone.0241705.s001.docx]

**S1 Table. Traditional ethnoveterinary uses of plants reported by the indigenous communities and local herbal practitioners of Punjab province.**

| **Tribe** | **S. #** | **Botanical name** | **Local name** | **Life trend** | **Part used** | **Preparation** | **Ethnoveterinary uses** |
| --- | --- | --- | --- | --- | --- | --- | --- |
| Andropogoneae | 1. | *Apluda mutica* L.  UOG-110 | Tachuli | Perennial | Aerial | Extract | Stomachache |
|  | 2. | *Bothriochloa bladhii* (Retz.) S.T. Blake  UOG-111 | Palvan | Perennial | Aerial | Decoction | Indigestion |
|  | 3. | *Chrysopogon aucheri* (Boiss.) Stapf  UOG-117 | Beerankha | Perennial | Leaves | Paste | Digestive disorders, improve fertility in bull |
|  | 4. | *Chrysopogon serrulatus* Trin.  UOG-118 | Chita Gha | Perennial | Leaves | Fodder | Antifungal & tonic |
|  | 5. | *Chrysopogon zizanioides* (L.) Roberty  UOG-122 | Vetiver, Khuss | Perennial | Leaves | Fodder | Antibacterial, anti-inflammatory |
|  | 6. | *Cymbopogon citratus* (DC) Stapf  UOG-131 | Lemon ghaas | Perennial | Leaves, seeds | Herbal tea | Headaches, tonic body, nervous system, stimulate glandular secretions |
|  | 7. | *Cymbopogon commutatus* (steud) Stapf.  UOG-133 | Lemon grass | Perennial | Whole | Extract | Indigestion, gastro enteritis |
|  | 8. | *Cymbopogon jwarancusa* (Jones.) Schult  UOG-134 | Khavi, Kittran | Perennial | Whole | Decoction | Typhoid fever, reproductive disorders |
|  | 9. | *Cymbopogon martini* (Roxb.) W. Watson  UOG-140 | Rauns | Perennial | Whole | Oil | Cough, fever, phlegmatic pains |
|  | 10. | *Dichanthium annulatum* (Forssk.) Stapf  UOG-141 | Dab Ghaah | Perennial | Whole | Paste | Indigestion |
|  | 11. | *Dichanthium foveolatum* (Delile) Roberty  UOG-145 | Humre | Perennial | Whole | Smoke | Smoke of plant is supposed useful to treat measles, bone healing |
|  | 12. | *Eulaliopsis binata* (Retz) C.E.Hubb.  UOG-147 | Sabai grass | Perennial | Stem | Decoction | Respiratory infections, fever, infectious, phlegmatic pains |
|  | 13. | *Heteropogon contortus* (L.) P Beauv. ex. Roem & Schult  UOG-151 | Kana | Perennial | Leaves | Extract, paste | Leucorrhoea, digestive disorders, anemia, typhoid |
|  | 14. | *Imperata cylindrica* (L.) Raeuschel  UOG-155 | Siru | Perennial | Leaves | Paste | Astringent, febrifuge, antibacterial |
|  | 15. | *Saccharum arundinaceum* Retz.  UOG-158 | Sarkanda | Perennial | Leave | Juice | Diuretic, refrigerant, diaphoretic, urinary  complaints, blood pressure |
|  | 16. | *Saccharum bengalense* Retz.  UOG-161 | Kana, Sarkanda | Perennial | Leaves | Juice | Oral infections dyspepsia, fever, constipation, hepatitis |
|  | 17. | *Saccharum spontaneum* L  UOG-165 | Kaa, Kahi | Perennial | Leaves, roots | Juice | Relieve in inflammation, urinary problems, treatment of abdominal pain, improvement of appetite |
|  | 18. | *Saccharum ravennae* L.  UOG-167 |  | Perennial | Aerial | Decoction | Typhoid, improvement of appetite, phlegmatic pains |
|  | 19. | *Saccharum officinarum* L.  UOG-169 | Gana | Perennial | Aerial | Juice | Improvement of appetite, diuretic, digestive disorders |
|  | 20. | *Sorghum bicolor* (L.) Moench  UOG-172 | Jowar, milo | Perennial | Aerial | Fodder, extract | Digestive problems |
|  | 21. | [*Sorghum saccharatum*](https://www.itis.gov/servlet/SingleRpt/SingleRpt?search_topic=TSN&search_value=521833)(L.) Moench  UOG-175 | Milo | Annual | Aerial | Extract | Diuretic, demulcent, serious abdominal pain, piles, malaria |
|  | 22. | *Sorghum halepense* (L.) Pers.  UOG-177 | Baru | Perennial | Root, whole | Fodder, extract | Infectious diseases |
|  | 23. | *Themeda anathera* (Nees) Hack  UOG-179 | Loonder, Lunji | Perennial | Leaves | Fodder, juice | Body cooling, depression, nervous exhaustion, shingles, herpes, refrigerant |
|  | 24. | *Themeda triandra* Forsk.  UOG-181 | Ghaa | Perennial | Leaves | Fodder | Anti-allergic, piles, malaria |
|  | 25. | *Vetiveria zizanioides* (L.) Nash  UOG-186 | Vetiver | Perennial | Leaves, seed | Decoction | Stomach problem, antiseptic, anti-inflammatory, demulcent |
|  | 26. | *Zea mays* L.  UOG-186 | Makai | Annual | Aerial | Paste, extract | Detoxifier, nerve tonic, antiseptic |
| Aristideae | 27. | *Aristida adscensionis* L.  UOG-188 | Lumb gaah | Annual | Aerial | Paster | Skin disorders |
|  | 28. | *Aristida cyanantha* Nees ex Steud.  UOG-191 | JangliGha | Annual | Aerial | Powder | Diuretic, anti-septic, anti-inflammatory, demulcent |
|  | 29. | *Aristida funiculata* Trin. & Rupr.  UOG-212 | Lumb | Annual | Leaves | Extract | Hypertension, hysteria, premature ejaculation, cure cancer, anti-fungal |
|  | 30. | *Aristida hystricula* (Edgew)  UOG-216 | Lumb | Annual | Aerial | Extract | Diuretic, antiseptic, blood pressure, fever, to treat dysfunctional organs of cattle, clear menstrual discharge |
|  | 31. | *Aristida mutabilis* Trin. & Rupr.  UOG-219 | Lumb | Annual | Leaves | Decoction, extract | Stomach problem, antiseptic, anti-inflammatory, piles |
|  | 32. | *Stipagrostis plumosa* (L.) Munro ex T.Anderson  UOG-233 | Chita gah | Annual | Whole | Fodder | Anticancer, sexual disorder |
| Arundineae | 33. | *Arundo donax* L.  UOG-236 | Nara bans, Nal, Narki | Perennial | Leaves, stem | Decoction | Blood pressure, fever, dysfunctional organs of cattle |
|  | 34. | *Phragmites australis*  (Cav.) Trin. ex Steud.  UOG-238 | Dila, Babyoon | Perennial | Leaves, root | Fodder, Powder | Digestive disorders, vomiting, bronchitis, cholera, diarrhea, cough, urinary tract infections |
|  | 35. | *Phragmites karka* (Retz.) Trin. ex Steud.  UOG-240 | Narr | Perennial | Leaves | Fodder, extract | Cardiac problem, antiemetic, detoxifier, shingles |
| Aveneae | 36. | *Avena fatua* L.  UOG-243 | Jangli Jai | Annual | Whole | Fodder | Stomach problem, cooling, styptic depression, nervous exhaustion, piles, |
|  | 37. | *Agrostis gigantea* Roth.  UOG-245 | Lamba gaah | Perennial | Leaves | Fodder | Anti-allergic, shingles, herpes |
|  | 38. | *Agrostis viridis* Gouan  UOG-247 | Forssk. | Perennial | Leaves | Decoction | Detoxifier, diaphoretic, diuretic, Hysteria |
|  | 39. | *Avena sativa* L.  UOG-260 | Jai, Wild Oats | Perennial | Aerial | Fodder | Detoxifier, piles, refrigerant, demulcent |
|  | 40. | *Avena sterilis* (Dur.) Gill & Magne  UOG-262 | Crazy oat | Annual | Aerial | Fodder | Diarrhea, dyspepsia, gastrointestinal disease, depression, herpes, hysteria |
|  | 41. | *Koeleria argentina* Griseb.  UOG-264 | Koeleria | Perennial | Leaves | Decoction | Treat sores and skin problems, anti-inflammatory |
|  | 42. | *Phalaris minor* Retz*.*  UOG-266 | Dum bisitti | Annual | Leaves | Decoction | Animal cough, depression, diaphoretic |
|  | 43. | *Polypogon monspeliensis* (L.) Desf.  UOG-268 | Malhar,  Dumbi citi | Annual | Aerial | Fodder | Cardiac disorders |
|  | 44. | *Trisetum clarkei* (Hook. f.) R. R. Stewart  UOG_271 |  | Perennial | Leaves | Extract | Relieve in inflammation, urinary problems, laxative |
|  | 45. | *Polypogon fugax* Nees ex Steud  UOG-274 | Beard grass | Annual | Stem | Juice, decoction | Improvement of appetite, diuretic, anti-inflammatory |
| Bromeae | 46. | *Bromus catharticus* Vahl  UOG-277 | Rescue grass | Annual | Leaves | Fodder | Improvement of appetite, diuretic, digestive disorders, cooling effect, anemia |
| Bambuseae | 47. | *Bambusa glaucescens* (Willd.) Merr.  UOG-279 | Bans | Perennial | Aerial | Paste, extract | Help to cure wounds, anemia, constipation, anti-allergic |
| Bromeae | 48. | *Bromus japonicus* Thunb.  UOG-281 | Silai ghass | Annual | Aerial | Fodder | Treat constipation, anti-toxin, relieve in inflammation |
|  | 49. | *Bromus pectinatus* Thunb.  UOG-286 | Chess grasses | Annual | Aerial | Fodder | Infusions used to normalize, increased heart palpitations |
|  | 50. | *Bromus sericeus* Drobov  UOG-289 | Brome grass | Perennial | Leaves | Fodder | To treat dysfunctional organs of cattle, diarrhea, anti-allergic |
| Chlorideae | 51. | *Tetrapogon cenchriformis* (A. Rich.) Clayton  UOG-292 |  | Annual | Leaves | Fodder | Diarrhea, dyspepsia, antiseptic & tonic, piles, anti-allergic |
|  | 52. | *Tetrapogon tenellus* (Roxb.) Chiov.  UOG-294 | Dumbi seeti | Annual | Leaves, Root | Fodder | Antibacterial, antifungal, diarrhea, dyspepsia, shingles |
|  | 53. | *Tetrapogon villosus* Desf.  UOG-297 | Sager | Perennial | Aerial | Powder, fodder | Treatment of abdominal pain, anti-bacterial |
| Cynodonteae | 54. | *Chloris gayana* Kunth  UOG-301 |  | Perennial | Leaves | Decoction | Treat constipation, diarrhea, anti-allergic, heart palpitations |
|  | 55. | *Chloris barbata* Sw.  UOG-305 | Jungle boti | Perennial | Aerial | Decoction | Diarrhea, dyspepsia, anti-inflammatory, styptic |
|  | 56. | *Chloris dolichostachya* Lag.  UOG-307 |  | Perennial | Aerial | Extract | Diabetic, diuretic, laxative, cough |
|  | 57. | *Chloris virgata* Sw.  UOG-308 | Boti | Perennial | Leaves | Paster, decoction | Diabetic, fracture, menstrual discharge, dysfunctional organs |
|  | 58. | *Cynodon dactylon* (L.) Pers.  UOG-309 | Khabbal, Tala | Perennial | Leaves | Paste, juice | Eye pain, skins injuries or cutting, anti-inflammatory, anemia, dysentery, heal bone fracture |
|  | 59. | *Cynodon radiates* Roth*.*  UOG-312 | Talar | Perennial | Whole | Decoction | Eyeache, relieve the eye pain, anti-inflammatory, haemostatic |
| Danthonieae | 60. | *Schismus arabicus* Nees  UOG-315 | Saryalaghas | Annual | Whole | Fodder, juice | Diuretic, laxative, cough, anti-toxin, demulcent |
| Eragrostideae | 61. | *Acrachne racemosa* (Heyne ex Roth) Ohwi  UOG-318 | Chinki | Annual | Whole | Fodder, paste | Skins injuries or cutting, controls dysentery, treat wounds, kidney problems, bronchial disorders |
|  | 62. | *Aeluropus lagopoides* (L.) Thwaites  UOG-320 | Kalar ghaa | Perennial | Whole | Fodder, juice | Haemostatic, antibiotic, anti-inflammatory, phlegmatic pains, relieve in inflammation |
|  | 63. | *Dactyloctenium* aristatum Link.  UOG-332 | Madhana ghaa | Annual | Leaves | Decoction | Laxative, cough, haemostatic, anti-allergic, herpes |
|  | 64. | *Dactyloctenium aegyptium* (L.) Wild.  UOG-338 | Koora, Madanah, | Annual | Whole | Decoction | Abdominal pains, malaria, haemostatic, anti-allergic, demulcent, detoxifier |
|  | 65. | *Dactyloctenium scindicum* Boiss.  UOG-342 | Dela | Perennial | Whole | Paste, fodder | Dysentery, jaundice, digestive disorders, anti-inflammatory |
|  | 66. | *Desmostachya bipinnata* L. Stapf  UOG-344 | Kusa, Dab | Perennial | Aerial | Decoction | Digestive disorders, diuretic, anti-amenorrhea |
|  | 67. | *Eragrostis amabilis* (L.) Wight & Arn.  UOG-349 | Love grass | Annual | Whole | Fodder, juice | Treatment of abdominal pain, kidney problems, clear menstrual discharge |
|  | 68. | *Eragrostis atrovirens* (Desf.) Trin. ex Steud.  UOG-355 | Thalia Grass | Perennial | Whole | Fodder, paste | Malaria, jaundice, anemia, dysentery, toothache |
|  | 69. | *Eragrostis barrelieri* Dav.  UOG-368 | Makni | Annual | Whole | Fodder | Diuretic, constipation |
|  | 70. | *Eragrostis ciliaris* (L.) R. Br  UOG-369 | Makni | Annual | Whole | Fodder, extract | Cure digestive disorders, astringent, detoxifier |
|  | 71. | *Eragrostis cilianensis* (All.) Janch.  UOG-373 | Stink grass | Annual | Whole | Fodder, juice | Digestive disorders, malaria, anti-allergic, herpes |
|  | 72. | *Eragrostis japonica* (Thunb.) Trin.  UOG-376 | Pan ghas | Annual | Leaves | Paste, fodder | Treat wounds, diuretic, anti-inflammatory |
|  | 73. | *Eragrostis pectinacea.* (Michx.) Nees  UOG-377 | Tufted grass | Annual | Whole | fodder | Urinary problems, laxative, gastrointestinal disease |
|  | 74. | *Eragrostis minor* Host.  UOG-379 | Choti ghas | Annual | Whole | fodder | Digestive disorders, anti-inflammatory, demulcent |
|  | 75. | *Eragrostis pilosa* (L.) P. Beauve  UOG-383 | Nika sanwak | Annual | Whole | Paste, decoction | Dysentery, toothache |
|  | 76. | *Eragrostis papposa* (Roem. & Schult.) Steud.  UOG-386 | Ghaa | Perennial | Aerial | Fodder | Controls itching, diuretic, constipation, jaundice, styptic |
|  | 77. | *Leptochloa panicea* (Retz.) Ohwi  UOG-388 | Paja | Annual | Whole | Fodder | Blood pressure, diuretic, constipation, anti-inflammatory |
|  | 78. | *Leptochloa chinensis* (L.) Nees  UOG-390 | Naru | Annual | Whole | Fodder | Diuretic, digestive disorders, sore, anti-allergic, homeostatic |
|  | 79. | *Eleusine indica* (L.) Gaertn  UOG-394 | Chezi, UntKatara | Annual | Aerial | Grain flour | Abdominal pain |
|  | 80. | *Sporobolus virginicus*(L.) Kunth  UOG-398 |  | Perennial | Whole | Fodder | Controls itching, treatment of abdominal pain, herpes, to treat dysfunctional organs |
| Paniceae | 81. | *Sporobolus nervosa* (Hocshsst.)  UOG-401 | Lambi ghaa | Perennial | Aerial | Fodder | Malaria, jaundice, anemia, dysentery, toothache |
| Eragrostideae | 82. | *Dactyloctenium aristatum* LinkUOG-403 | chhaibbnrr | Perennial | Whole | Decoction | Diuretic, constipation |
|  | 83. | *Dactyloctenium scindicum* Boiss  UOG-406 | Crow foot | Perennial | Whole | Fodder, paste, decoction | Cure digestive disorders, astringent, wounds treatment |
| Hainardeae | 84. | *Parapholis strigosa* (Dum.) C. E. Hubbard  UOG-408 | Tooti ghas | Perennial | Whole | fodder | Leucorrhoea, digestive disorders, malaria, anti-allergic, relieve in inflammation |
| Oryzeae | 85. | *Oryza sativa* L.  UOG-409 | Chawal | Annual | Aerial | Paste, decoction | Diarrhea, wound healing |
| Pappophoreae | 86. | *Enneapogon shimpranus* (Hochst. ex A. Rich) Renvoize  UOG-412 | Jeo | Perennial | Whole | Fodder, paste | Disinfectant, digestive disorders, anti-allergic |
|  | 87. | *Enneapogon persicus* Boiss.  UOG-416 | Jiu, Sabri | Perennial | Whole | Fodder | Improves digestion, laxatives, anti-fungal, controls itching |
|  | 88. | *Enneapogon desvauxii* P. Beauv.  UOG-418 | Dhui | Annual | Leaves, seed | Fodder | Digestive disorders, malaria |
| Paniceae | 89. | *Digitaria nodosa* Parl.  UOG-421 | Swank | Perennial | Whole | Fodder | Digestive disorders, malaria, laxatives, anti-bacterial |
|  | 90. | *Brachiaria distachya* (L.) Stapf.  UOG-423 | Jangli ghas | Annual | Whole | Fodder, juice | Jaundice, anti-allergic, detoxifier, clear menstrual discharge, General weakness |
|  | 91. | *Brachiaria deflexa* (Schumach.) C.E.Hubbard ex Robyns  UOG-427 | Moti ghas | Annual | Whole | Fodder, powder | Kidney problems, anti-inflammatory, styptic, demulcent, herpes |
|  | 92. | *Brachiaria mutica* (Forssk.) Stapf.  UOG-429 | Bubbr Kha | Annual | Leaves | Fodder, extract | Toothache, sore, anti-inflammatory, controls itching |
|  | 93. | *Brachiaria eruciformis* (sm) Griseb.  UOG-433 | Sawari, Jhanda | Annual | Whole | Juice, paste | Leaves work as antiseptic, relieve in inflammation |
|  | 94. | *Brachiaria adspersa* (Trin.) Parodi  UOG-437 | Hausa, Sair | Annual | Stem | Juice | Leaves juice helps to cure anemia, laxatives, diuretic |
|  | 95. | *Brachiaria ovalis* Stapf  UOG-439 | Ghaah | Perennial | Leaves | Juice | Anti-inflammatory |
|  | 96. | *Cenchrus biflorus* Roxb.  UOG-441 | Bhurat | Annual | Aerial | Extract | Urinary problems |
|  | 97. | *Cenchrus ciliaris* L.  UOG-444 | Dhaman | Perennial | Aerial | Juice, extract | Urinary disorders |
|  | 98. | [*Cenchrus prieurii* (Kunth) Maire](http://www.theplantlist.org/tpl1.1/record/kew-403998)  UOG-448 | Dhaman | Annual | Leaves | Juice | Digestive disorders, anemia, toothache, sore, general weakness |
|  | 99. | *Cenchrus pennisetiformis* Steud.  UOG-454 | Bara Dhaman | Annual | Whole | Paste, powder | Kidney problems, digestive disorders, herpes |
|  | 100. | *Cenchrus setiger* Vahl**.**  UOG-458 | Kala dhaman, Talra | Perennial | Aerial | Extract, powder | Disinfectant, kidney pain, sore, wound, phlegmatic pains |
|  | 101. | *Digitaria pennata* (Hochst.) T.Cooke  UOG-459 | Tera | Perennial | Leaves | Fodder | Kidney problems, digestive disorders, anti-allergic |
|  | 102. | *Digitaria ciliaris* (Retz.) Koeler  UOG-462 | Shamokha, Tandla | Annual | Whole | Fodder | Diuretic, aphrodisiac, digestive disorders, anti-fungal |
|  | 103. | *Digitaria longiflora* (Retz.) Pers.  UOG-465 | Deeta, Indian Crab Grass | Annual | Whole | Fodder | Diuretic, kidney pain, general weakness, stimulant, cure wounds, styptic |
|  | 104. | *Digitaria stricta* Rotch  UOG-468 | Meru | Perennial | Leaves | Fodder | Treat tumors, diuretic, emollient, malaria, digestive disorders, Anti-allergic |
|  | 105. | *Digitaria radicosa* (Presl) Miq.  UOG-477 | Trilling Crab Grass | Perennial | Whole | Fodder | Leucorrhoea, digestive disorders, kidney pain, tonic |
|  | 106. | *Digitaria sanguinalis* (L.) scop.  UOG-485 | GhandhalaGhaa | Annual | Whole | Fodder | Antiseptic, stimulant, treat tumors, kidney pain, sore |
|  | 107. | *Digitaria setigera* Roth.  UOG-489 | Ungli Gha, Fonio | Perennial | Whole | Fodder | Laxatives, diuretic, malaria, tonic, refrigerant, clear menstrual discharge |
|  | 108. | *Digitaria violascens* Link.  UOG-490 | Violet Crab Grass | Perennial | Leaves, root | Fodder | Stimulant, treat tumors, diuretic, anti-bacterial, herpes |
|  | 109. | *Echinochloa colona* (L.) Link  UOG-493 | Jungli chowol,  Sanawakri | Annual | Whole | Paste | Digestive disorders, general weakness, constipation, phlegmatic pains |
|  | 110. | *Echinochloa crus-galli* (L.) P. Beauv.  UOG-495 | Sanwak | Annual | Whole | Juice | Digestive disorders, demulcent |
|  | 111. | *Ochthochloa compressa* (Forssk.) Hilu.  UOG-497 | Gandeel | Perennial | Aerial | Grains, raw-material | Kidney pain, anti-allergic, clear menstrual discharge |
|  | 112. | *Panicum antidotale* Retz. UOG-499 | Gharam, Morrot | Perennial | Whole | Decoction, Juice | Anti-bacterial |
|  | 113. | *Panicum atrosanguineum* Hochst. Ex A. Rich  UOG-500 | Moti Gha | Perennial | Seed | Decoction | Fever, diuretic, tonic, laxative, wounds treatment, gastrointestinal disease |
|  | 114. | *Panicum maximum* Jacq.  UOG-501 | Bansi Gha | Perennial | Whole | Paste, decoction | Digestive disorders, diuretic, tonic, malaria, emollient |
|  | 115. | *Panicum turgidum* Forssk  UOG-505 | Bansi | Perennial | Whole | Paste | Diuretic, febrifuge, tonic, Skin disorders, anti-allergic |
|  | 116. | *Panicum sumatrense* Roth.  UOG-507 | Cheena | Perennial | Leaves | Juice, paste | Diuretic, laxative, cure cancer, gastrointestinal diseases |
|  | 117. | *Paspalidium geminatum* (Forssk.) Stapf  UOG-511` | Knot grass | Perennial | Whole | Fodder, Juice | Diuretic, laxative, anti-bacterial, to treat dysfunctional organs, general weakness |
|  | 118. | *Paspalidium flavidum* (Retz.) A. Camus  UOG-513 | Gandh ghas | Perennial | Leaves | Powder | Urinary problems, laxative, anti-allergic, demulcent |
|  | 119. | *Paspalidium punctatum* (Burm.) A. Camus  UOG-524 | Nseila | Perennial | Whole | Paste, decoction | Kidney problems, diuretic, oral infections, anti-bacterial |
|  | 120. | *Paspalum paspaloides* (Michx.) Scribner.  UOG-533 | Maro | Perennial | Leaves | Juice | Digestive disorders, antibiotic |
|  | 121. | *Panicum repens* L*.*  UOG-539 | Goli gha | Perennial | Whole | Juice | Digestive disorders, wound healing, emollient, treatment of dysfunctional organs |
|  | 122. | *Pennisetum divisum* (Forssk. ex J.F.Gmel.) Henrard UOG-542 | Awansi grass | Perennial | Leaves | Decoction | Urinary problems, anti-inflammatory, oral infections |
|  | 123. | *Pennisetum glaucum* (L.) R.Br.  UOG-546 | Bhaajhri | Perennial | Whole | Extract | Digestive disorders, jaundice, antibiotic, wound healing |
|  | 124. | *Pennisetum americanum* (L.) Leeke  UOG-555 | Bajra | Perennial | Leaves, root | Decoction, juice | Digestive disorders, jaundice, detoxifier |
|  | 125. | *Pennisetum orientale* Rich  UOG-566 | Haathi ghaa | Perennial | Whole | Decoction | Relieve in inflammation |
|  | 126. | *Sporobolus ioclados* (Nees. ex. Trin.) Nees.  UOG-567 | Swag | Perennial | Leaves, root | Fodder | Remove debris from wounded area, anti-allergic, general weakness |
|  | 127. | *Paspalum dilatatum* Poir.  UOG-577 | Batto | Perennial | Leaves | Juice | Laxative, gastrointestinal disease, oral infections |
|  | 128. | *Setaria glauca* (L.) P. Beauv  UOG-578 | Ban Kangni | Annual | Whole | Extract | Leucorrhoea, anemia, skin disorders, digestive disorders |
|  | 129. | *Setaria intermedia* Roem. & Schult.  UOG-584 | Chirchira | Annual | Whole | Powder, juice | Diabetes, gastrointestinal disease, oral infections |
|  | 130. | *Setaria italica* (L.) P.Beauv.  UOG-587 | Kangni | Annual | Leaves | Decoction, extract | Urinary problem, anti-bacterial, relieve in inflammation |
|  | 131. | *Setaria pumila* (Poir) Roem. & Schult.  UOG-589 | Ban kangni | Annual | Aerial | Extract | Oral infections, general weakness |
|  | 132. | *Setaria verticillata* (L.) P. Beauv.  UOG-590 | Barchittas | Annual | Leaves | Grains | Indigestion demulcent |
|  | 133. | *Setaria viridis* (L.) P. Beauv.  UOG-593 | Kangni | Annual | Leaves, stem | Grains | Urinary problems, febrifuge and tonic. |
|  | 134. | *Brachiaria prostrata* (Lam) Griseb  UOG-596 | Common Sandbur | Annual | Leaves | Juice | Headaches, tonic body, nervous system |
|  | 135. | *Brachiaria ramosa* (L.) Stapf  UOG-600 | Brown top Millet | Perennial | Leaves | Juice | Infectious diseases, indigestion, laxative, anti-allergic |
|  | 136. | *Pennisetum lansatum* Klotzsch.  UOG-605 | Awansi grass | Annual | Whole | Fodder | Diuretic, improve fertility in bull, oral infections |
|  | 137. | *Urochloa panicoides* P. Beauv.  UOG-611 | Harat | Annual | Leaves | Smoke | Smoke of plant is supposed useful to treat measles, febrifuge, sore |
|  | 138. | *Urochloa setigera* (Retz.) Stapf.  UOG-615 | Jhun | Annual | Whole | Decoction | Typhoid fever, diabetes, demulcent |
| Poeae | 139. | *Dactylis glomerata* L.  UOG-621 | Gadu | Perennial | Leaves | Extract | Diuretic, sore, herpes |
|  | 140. | *Lolium temulentum* L.  UOG-625 | Cockle | Perennial | Leaves | Grains | Nervous disorders, to treat dysfunctional organs |
|  | 141. | *Lolium persicum* Boiss. & Hohen.  UOG-631 | Bera ghas | Annual | Leaves | Grains | Respiratory infections, fever, diabetes, to treat dysfunctional organs, improve fertility in bull, general weakness |
|  | 142. | *Poa annua* L.  UOG-633 | Blue Grass, Jaie | Annual | Whole | Fodder, decoction | Gastrointestinal disease |
|  | 143. | *Poa infirma* Kunth.  UOG-637 | Wakh, Kandail | Annual | Whole | Decoction, juice | Digestive disorders, jaundice, diabetes, anti-fungal |
| Triticeae | 144. | *Hordeum vulgare* L.  UOG-645 | Jao | Annual | Leaves, seed | Paste, powder | Leucorrhoea, anemia, skin cleaner, diabetes, herpes |
|  | 145. | *Triticum aestivum* L.  UOG-648 | Kanak, Gandum | Annual | Whole | Paste | Anti-cancerous, gastrointestinal disease |
| Zoysieae | 146. | *Leptothrium senegalense* (Kunth) Clayton  UOG-650 | Madhani | Annual | Whole | Juice, powder | Cure cancer, laxative, anti-inflammatory, anti-allergic |
|  | 147. | *Tragus berteronianus* Schult.  UOG-655 |  | Annual | Leaves | Decoction | Digestive disorders, nervous disorders, anti-bacterial |
|  | 148. | *Tragus racemosus* (L.) All.  UOG-666 | Swanri | Annual | Whole | Decoction | Digestive disorders, laxative, cure cancer, anti-allergic |
|  | 149. | *Tragus roxburghii* Panigrahi  UOG-786 | Bur grah | Annual | Whole | Juice | Laxative, gastrointestinal disease, diabetes, Cure cancer, anti-bacterial |
