# Supplementary material for: Ethno-veterinary uses of Poaceae in Punjab, Pakistan
Source: PLoS One. 2020 Nov 3;15(11):e0241705. doi: 10.1371/journal.pone.0241705 (PMC7608896; doi:10.1371/journal.pone.0241705)
Supplement: S2 Table — Correlation analysis between a) UV vs CSI, b) RFC vs SCI, and c) UV vs RFC. (DOC) [file pone.0241705.s002.doc]

		Table S2. The correlation analysis between. a) UV vs CSI, b) RFC vs SCI, and c) UV vs RFC 

		RFC	UV	CSI	
RFC	Pearson Correlation	1	.251**	.599**	
	Sig. (2-tailed)		.002	.000	
	N	149	149	149	
UV	Pearson Correlation	.251**	1	.517**	
	Sig. (2-tailed)	.002		.000	
	N	149	149	149	
CSI	Pearson Correlation	.599**	.517**	1	
	Sig. (2-tailed)	.000	.000		
	N	149	149	149	
**. Correlation is significant at the 0.01 level (2-tailed).	
