# Supplementary figures and images for: Ethno-veterinary uses of Poaceae in Punjab, Pakistan
Source: PLoS One. 2020 Nov 3;15(11):e0241705. doi: 10.1371/journal.pone.0241705 (PMC7608896; doi:10.1371/journal.pone.0241705)

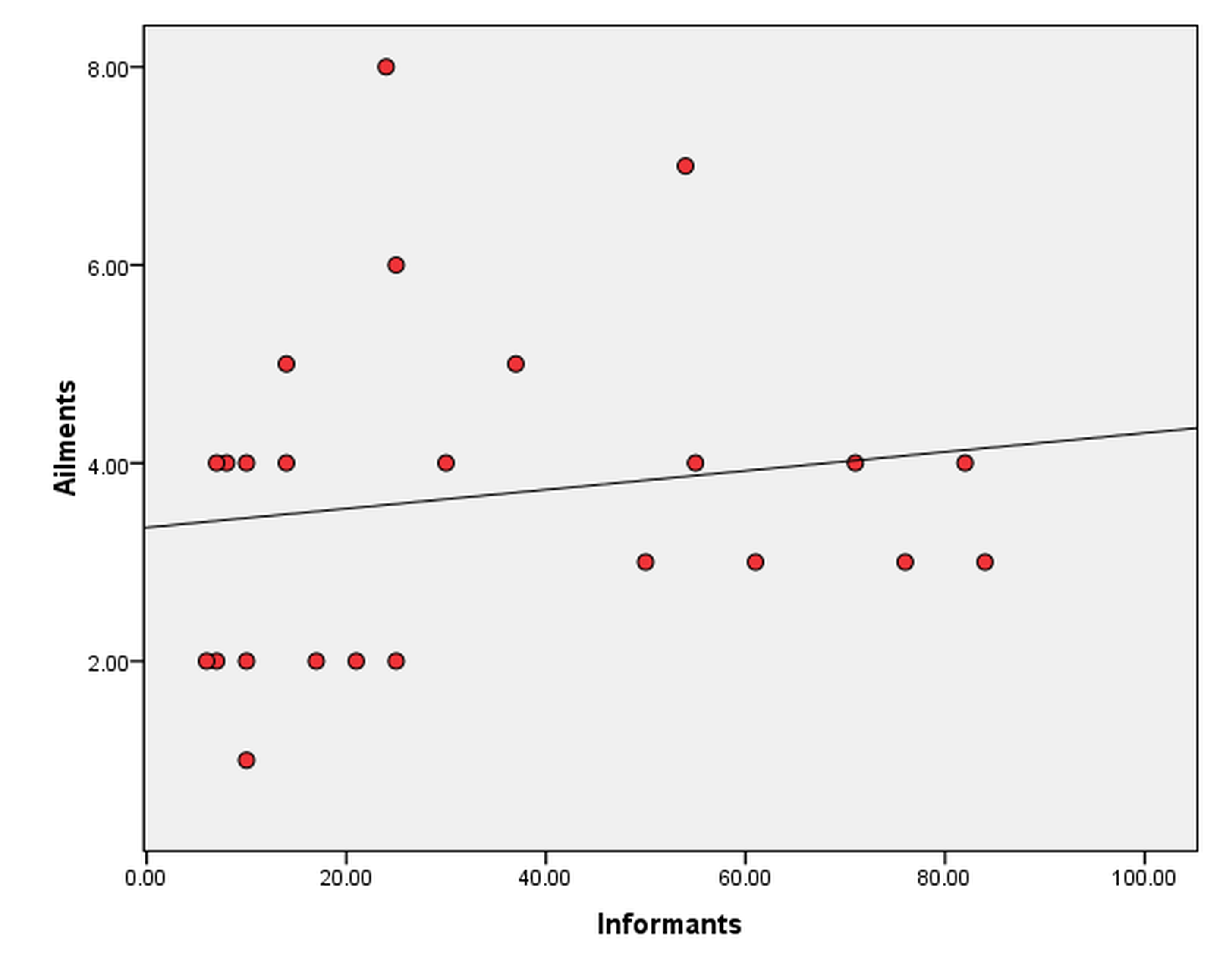

Supplement: S1 Fig — (TIF) [file pone.0241705.s003.tif]
